# Vancomycin-Induced Modulation of Gram-Positive Gut Bacteria and Metabolites Remediates Insulin Resistance in iNOS Knockout Mice

**DOI:** 10.3389/fcimb.2021.795333

**Published:** 2022-01-19

**Authors:** Hobby Aggarwal, Priya Pathak, Vishal Singh, Yashwant Kumar, Manoharan Shankar, Bhabatosh Das, Kumaravelu Jagavelu, Madhu Dikshit

**Affiliations:** ^1^ Pharmacology Division, Council of Scientific and Industrial Research (CSIR)-Central Drug Research Institute, Lucknow, India; ^2^ Department of Nutritional Sciences, The Pennsylvania State University, State College, PA, United States; ^3^ Non-Communicable Diseases Division, Translational Health Science and Technology Institute, Faridabad, India; ^4^ Microbial Physiology Laboratory, Department of Bioscience & Bioengineering, Indian Institute of Technology, Jodhpur, India; ^5^ Molecular Genetics Laboratory, Infection and Immunology Division, Translational Health Science and Technology Institute, Faridabad, India

**Keywords:** dyslipidemia, insulin resistance, obesity, gut microbiota, metabolome analysis, iNOS^−/−^ mice

## Abstract

The role of oxidative and nitrosative stress has been implied in both physiology and pathophysiology of metabolic disorders. Inducible nitric oxide synthase (iNOS) has emerged as a crucial regulator of host metabolism and gut microbiota activity. The present study examines the role of the gut microbiome in determining host metabolic functions in the absence of iNOS. Insulin-resistant and dyslipidemic iNOS^−/−^ mice displayed reduced microbial diversity, with a higher relative abundance of *Allobaculum* and *Bifidobacterium*, gram-positive bacteria, and altered serum metabolites along with metabolic dysregulation. Vancomycin, which largely depletes gram-positive bacteria, reversed the insulin resistance (IR), dyslipidemia, and related metabolic anomalies in iNOS^−/−^ mice. Such improvements in metabolic markers were accompanied by alterations in the expression of genes involved in fatty acid synthesis in the liver and adipose tissue, lipid uptake in adipose tissue, and lipid efflux in the liver and intestine tissue. The rescue of IR in vancomycin-treated iNOS^−/−^ mice was accompanied with the changes in select serum metabolites such as 10-hydroxydecanoate, indole-3-ethanol, allantoin, hippurate, sebacic acid, aminoadipate, and ophthalmate, along with improvement in phosphatidylethanolamine to phosphatidylcholine (PE/PC) ratio. In the present study, we demonstrate that vancomycin-mediated depletion of gram-positive bacteria in iNOS^−/−^ mice reversed the metabolic perturbations, dyslipidemia, and insulin resistance.

## 1 Introduction

Type 2 diabetes is a multitudinous metabolic disorder that arises from a complex interaction among genetic and environmental elements including dysregulated microbiota composition and function (Ahlqvist et al., 2018). Insulin resistance (IR) or dyslipidemia is a key attribute of obesity and diabetes due to metabolic disruptions (Ormazabal et al., 2018). Gut microbiota dysbiosis, specifically changes in Firmicutes to Bacteroidetes ratio, has been linked to a cluster of chronic metabolic diseases including IR, diabetes, and obesity in both humans and mice (Eid et al., 2017). Prebiotics (such as inulin), probiotics, and other microbiota-targeted bacteriotherapy including antibiotics interventions are actively being explored to prevent and manage metabolic disorders (Shapiro et al., 2017). Treatment with antibiotics reduces bacterial diversity in the host with reduced resistance to colonization of non-autochthonous microbes (Sullivan, 2001; Dethlefsen et al., 2008). Thus, antibiotics add an interesting dynamism to the host–microbiome association (Jernberg et al., 2010). Antibiotic-induced depletion of the short-chain fatty acid (SCFA)-producing gut microbes reduced the levels of microbial-derived colonic SCFA in children and enhanced the likelihood to develop obesity and metabolic syndrome later in life (Korpela et al., 2017; Li et al., 2017a; Wilkins and Reimer, 2021). On the contrary, antibiotic-induced microbiota depletion (AIMD) by the antibiotic cocktail or vancomycin provides protection against diet or genetically inherited obesity and glucose dysmetabolism (Carvalho et al., 2012; Hwang et al., 2015; Fujisaka et al., 2016) by reducing inflammation and oxidative stress and improving metabolic homeostasis (Cani et al., 2008). Studies in humans have implied both gram-positive and gram-negative bacteria in the metabolic perturbations and obesity (Rawat et al., 2012; Pushpanathan et al., 2016; Radilla-Vázquez et al., 2016; Liu et al., 2019). These studies thus indicate the strong association of gut microbiota with obesity and related metabolic disorders (Cox and Blaser, 2013).

Nitric oxide (NO) has a pivotal role in the regulation of cardiovascular and metabolic functions. Among the different NOS isoforms, the importance of iNOS has been predominantly investigated in inflammatory and infectious diseases (Wong and Billiar, 1995; Kröncke et al., 1998) but is now emerging as an important metabolic regulator (Perreault and Marette, 2001; Cha et al., 2011; Kanuri et al., 2017; Kakimoto et al., 2019; Pathak et al., 2019; Aggarwal et al., 2020). iNOS-derived NO controls the growth of certain microorganisms as an adaptive response of host defense, while survival of certain microbes also seems to be facilitated (Bogdan, 2015). iNOS^−/−^ mice are protected from LPS-induced IR (Carvalho-Filho et al., 2006) and cardiovascular (House Ii et al., 2012) and endothelial dysfunction (Chauhan et al., 2003) and also displayed reduced inflammatory cytokines (Perreault and Marette, 2001; Cha et al., 2011) with enhanced survival during septicemia (Hollenberg et al., 2000). Moreover, previous studies from our lab (Kanuri et al., 2017; Pathak et al., 2019; Aggarwal et al., 2020) and others (Nakata et al., 2008; Kakimoto et al., 2019) had demonstrated IR and disrupted metabolic homeostasis in iNOS^−/−^ mice fed with chow diet, low-fat diet (LFD), or high-fat diet (HFD) which was reversed by enhancing NO bioavailability *via* nitrite treatment (Aggarwal et al., 2020), suggesting the importance of redox status in host metabolism. As oxidative stress and changes in gut microbiota could play a role in IR (Cui et al., 2009), iNOS^−/−^ mice might have dysbiosis due to the absence of iNOS-derived NO (Aggarwal et al., 2020).

Therefore, our present study explored the contribution of gut microbiota in regulating the metabolic abnormality observed in mice lacking iNOS. We demonstrate that iNOS^−/−^ mice exhibit an altered gut microbial community with enhanced enrichment of gram-positive bacteria. Vancomycin (largely depletes gram-positive bacteria)-induced depletion of gut bacteria established that such enrichment of vancomycin-sensitive microbes is a vital contributor to metabolic abnormalities found in iNOS^−/−^ mice.

## 2 Materials and Methods

### 2.1 Mice and Diet

Twelve-week-old, age-matched male C57BL/6 (WT) and iNOS knockout (iNOS^−/−^) (Jackson Laboratory, USA; 002609) mice on C57BL/6J background were bred and maintained in IVC cages (Tecniplast, Italy) at 24°C ± 2°C. All procedures were approved by the Institutional Animal Ethics Committee of CSIR-CDRI (IAEC/2014/43) in accordance with CPCSEA guidelines. Mice (WT and iNOS^−/−^) were maintained on chow diet (1320, Altromin, Germany) and water *ad libitum*. The antibiotic vancomycin (0.5 g/l) and antibiotic cocktail (Abx) comprising of ampicillin (1 g/L), neomycin (1 g/l), metronidazole (1 g/L), and vancomycin (0.5 g/L) were administered *via* drinking water for 4 weeks for depletion of gram-positive anaerobic bacteria and majority of gut microbiota, respectively (Hansen et al., 2012; Rodrigues et al., 2017).

### 2.2 Body Weight, Body Mass Index, and Food Consumption

Body weight was measured weekly from day 0 to the completion of the study at 4 weeks. The body length was also measured from the nose tip to the base of the tail of each mouse. Body mass index (BMI) was calculated using the formula body weight (g) divided by the square of nose to anus length (cm) as previously described (Aggarwal et al., 2020) at the end of 4 weeks of antibiotic treatment. The weekly food consumption was measured by housing two to three mice per cage and adding the preweighed food pellets to each cage and measuring the food left over on a sensitive weighing balance twice weekly. The average weekly food consumption and the average food intake/day/mice during the study period were calculated.

### 2.3 Tolerance Tests

Mice were administered 2 g/kg D-glucose, 2 g/kg sodium pyruvate, or 0.6 IU/kg insulin (Human insulin R, Eli Lilly) by intraperitoneal (i.p.) route to perform glucose (GTT), pyruvate (PTT), or insulin tolerance test (ITT), respectively, after 6 h of fasting. Blood glucose was monitored using the Accu-Chek glucometer (Roche Diagnostics, India) at 0, 15, 30, 60, and 120 min after the administration of glucose, pyruvate, or insulin, and the area under the curve (AUC) was calculated as described previously (Kanuri et al., 2017).

### 2.4 Body Composition Analysis

Body composition (fat and lean mass) was analyzed in live, conscious mice by echo MRI (E26-226-RM Echo MRI LLC, USA) allowing limited horizontal and vertical movements by applying radiofrequency pulses at a distinct static magnetic field (Kanuri et al., 2018).

### 2.5 *In-Vivo* Gut Permeability Assay

FITC-labeled dextran (4 kDa) was used to assess *in-vivo* intestinal permeability (Thevaranjan et al., 2017). Mice were fasted for 4 h with free access to water. A total of 0.8 mg/ml FITC-dextran tracer was given orally in 200 µl PBS followed by removal of both food and water after the gavage. Blood was collected retro-orbitally after 4 h, the serum was separated, and fluorescence intensity was measured using an excitation wavelength of 493 nm and an emission wavelength of 518 nm.

### 2.6 Serum Biochemistry

Retro-orbital blood was collected from 6-h fasted mice. Estimation of lipids like total cholesterol (TC), triglycerides (TG), low- and high-density lipoproteins (LDL and HDL), and non-esterified fatty acids (NEFA) was performed in the serum using kits (Pathak et al., 2019) (Randox, UK). Insulin was measured using the kit from Crystal Chem, USA. Indices of IR (HOMA-IR) and insulin sensitivity (QUICKI) were calculated from fasting blood glucose and serum insulin as per the formulae used by other investigators (Yokoyama et al., 2003).

### 2.7 Total Nitrite Estimation

The animals were sacrificed to retrieve the tissues (liver, epididymal white adipose tissue, and small intestine). Tissues (liver, eWAT, and intestine, 50 mg) were homogenized in 500 µl of hypotonic TKM buffer [25 mM Tris–HCl (pH 7.4), 2 mM MgCl_2_, 5 mM KCl, and 1% NP-40] followed by sonication. The supernatant was obtained by centrifugation at 15,000*g* at 4°C for 20 min. Total nitrite (nitrate and nitrite) was estimated in serum (100 μl) and tissue homogenates using Griess reagent by reducing nitrate to nitrite using preactivated cadmium pellets followed by deproteinization in tissue homogenates with 3% trichloroacetic acid (Kanuri et al., 2017).

### 2.8 Tissue Biochemistry

Liver tissue (50 mg) was processed as described previously (Aggarwal et al., 2020) for the estimation of hepatic total cholesterol, triglycerides, and free fatty acids (FFA) using the Randox kit. Briefly, for TC estimation, samples were homogenized on ice in 1 ml hexane:isopropanol mixture (3:2 ratio) followed by centrifugation, supernatant collection, and drying in a CentriVap concentrator (Labconco, USA). Samples were homogenized in 1 ml of 1% Triton X-100 in chloroform on ice, centrifuged, and lower phase dried in CentriVap for FFA estimation. The dried products for TC and FFA estimation were redissolved in ethanol:NP-40 (9:1). For triglyceride estimation, samples were homogenized in 500 µl of 5% NP-40 on ice, boiled for 5 min in a water bath at 80°C–100°C, then cooled and reheated three times, and centrifuged, and the supernatant was collected.

### 2.9 Hepatic Glycogen

Insulin was administered at a dose of 0.6 IU/kg i.p. and the animals were sacrificed after 30 min to collect the liver for insulin-stimulated glycogen estimation along with unstimulated controls in both WT and iNOS^−/−^ mice with or without vancomycin and Abx treatment. A 50-mg liver tissue was homogenized in 500 µl distilled water on ice, boiled for 10 min, and centrifuged to remove insoluble substances followed by supernatant collection and glycogen estimation using the kit from Biovision (K646-100) as per the protocol of the manufacturer.

### 2.10 Alcian Blue (AB) Staining

Formalin-fixed, paraffin-embedded small intestinal and colonic tissue was sectioned into 5 μm thin serial slices and stained with 1% Alcian blue solution (in 3% acetic acid, pH 2.5) for morphological examination of acid mucosubstances and acetic mucins and counterstained with 0.1% nuclear Fast Red solution (Otsuka et al., 2010). The Alcian blue-stained area (%), villi, and crypt lengths were calculated using ImageJ software.

### 2.11 Real-Time PCR

Quantitative gene expression analysis was performed using SYBR Green as described previously (Kanuri et al., 2018). Briefly, total RNA was extracted using the TRIzol reagent followed by cDNA synthesis using RevertAid first-strand cDNA synthesis kit using the protocol of the manufacturer. Real-time PCR was performed using LightCycler 480II Real-Time PCR system (Roche Applied Science, Indianapolis, IN, USA) with primers listed in **Table S1**. 18S rRNA was used as a reference gene for normalization in liver and adipose tissues and RPL10 in intestinal tissue to calculate the expression of candidate genes. Relative fold change was calculated from mean normalized gene expression between different groups as compared with WT mice.

### 2.12 Metabolomics Analysis

#### 2.12.1 Sample Preparation

A 100-µl serum was lyophilized and stored at −80°C until further processing. The sample was reconstituted in 200 µl methanol, 50 µl water, and 870 µl methyl tert-butyl ether (MTBE) and vortexed for 1 h to extract the metabolites. Organic and aqueous phase separation was induced by adding 250 µl water and centrifuged at 15,000*g* for 15 min at 4°C. The upper organic and lower aqueous layers (100 µl each) were vacuum dried in a SpeedVac concentrator and stored until further analysis at −80°C. Samples were reconstituted in 50 µl 15% methanol and kept on ice for 30 min, vortexed for another 30 min, and centrifuged at 15,000*g* for 15 min at 4°C, and the supernatant was collected and subjected to metabolomic analysis by using the LC-MS platform.

#### 2.12.2 Metabolomics Measurement

The metabolomics data were acquired on the Orbitrap fusion mass spectrometer (Thermo Scientific, USA) equipped with a heated electrospray ionization (HESI) source. Data were acquired on positive and negative modes at 120,000 mass resolution in MS mode and 30,000 resolution in data-dependent MS2 scan mode. Spray voltages of 4,000 and 35,000 V were used for the positive and negative modes, respectively. Sheath gas and auxiliary gas was set to 42 and 11, respectively. Mass scan range of 50–1,000 *m*/*z*, automatic gain control (AGC) target at 200,000 ions, and maximum injection time of 80 ms for MS and AGC target of 20,000 ions and maximum injection time of 60 ms for MSMS were used. The extracted metabolites were separated on UPLC ultimate 3000 using HSS T3 column (100 × 2.1 mm i.d., 1.7 µm, waters) maintained at 40°C temperature. Mobile phase A was water with 0.1% formic acid and mobile phase B was acetonitrile with 0.1% formic acid. The elution gradient used is as follows: 0 min, 1% B; 1 min, 15% B; 4 min, 35% B; 7 min, 95% B; 9 min, 95% B; 10 min, 1% B; and 14 min, 1% B. The flow rate was 0.3 ml/min and the sample injection volume was 5 µl. The pool quality control (QC) sample was prepared by collecting 10 µl from each sample and was run after every five samples to monitor retention time shift, signal variation, and drift in mass error (Kumar et al., 2020).

#### 2.12.3 Data Processing

All acquired data were processed using the Progenesis QI software (Waters Corporation) using default setting. The untargeted metabolomics workflow of Progenesis QI was used to perform retention time alignment, feature detection, elemental composition prediction, and database search. Identification of the metabolite was done on the basis of an in-house metabolite library with accurate mass, retention time, and fragmentation pattern information match. Additionally, spectral data matching with mzCloud and MassBank for the fragmentation match for the identification of metabolites were also used. Metabolomics data were normalized by sum and Pareto scaled before multivariate analysis. Relative fold change values in metabolite expression analysis were calculated for each treated sample with respect to the untreated time-matched control (WT) for two sets of experiments, and then differential analysis was performed on the pooled fold change data. Fold change values were log transformed for a clearer representation in the heatmap analysis. Statistical analysis was performed by multiple *t*-tests followed by a two-stage linear step-up procedure of Benjamini, Krieger, and Yekutieli to correct for multiple comparisons by controlling the false discovery rate (<0.05).

### 2.13 16S rRNA Gene Sequence Analysis

Genomic DNA from approximately 200 mg mice stool samples was extracted by an optimized enzymatic, chemical, physical, and mechanical lysis method as described previously (Bag et al., 2016). The V3–V4 region of the *16S rRNA* gene was amplified and gel extracted for metagenomic sequencing as described previously (Tandon et al., 2018). Metagenome libraries were prepared using V3–V4 region-specific primers. Approximately, 40 ng of DNA samples were amplified for 26 cycles of round 1 PCR using KAPA HiFi Hot-Start PCR Kit (KAPA Biosystems Inc., USA). The forward and reverse primers were used at a concentration of 5 µM each. The amplicons were analyzed on 1.2% agarose gel, and l µl of diluted round 1 PCR amplicons was used for indexing PCR (round 2). Here, the round 1 PCR amplicons were amplified for 10 cycles to add Illumina sequencing barcoded adaptors (Nextera XT v2 Index Kit, Illumina, USA). Round 2 PCR amplicons (sequencing libraries) were analyzed on 1.2% agarose gel. The Illumina adapter sequences were as follows: 5′-AATGATACGGCGACCACCGAGATCTACAC[i5]TCGTCGGCAGCGTC and 5′-CAAGCAGAAGACGGCATACGAGAT[i7]GTCTCGTGGGCTCGG, where [i5, i7] were unique dual index sequence to identify sample-specific sequencing data. Furthermore, the samples were loaded into an Illumina MiSeq v3 600 cycles flow cell (Illumina, CA, USA) and the sequencing run was performed according to standard Illumina protocol.

#### 2.13.1 Data Analysis

The Illumina paired-end raw reads having V3–V4 primer sequence and high-quality bases (>Q30) were selected. Bcl2fastq software v2.20 was used for data demultiplexing and FastQ files were generated based on the unique dual barcode sequences. FastQC v0.11.8 software was used to assess the sequencing quality. The adapter sequences were trimmed and bases above Q30 were considered. During read preprocessing, low-quality bases were filtered off and then data were used for downstream analysis. The reads were further stitched using Fastq-join. These stitched reads were subjected to QIIME pipeline for microbiome analysis. The query sequences were clustered using the UCLUST method against a curated chimera-free 16S rRNA database, Greengenes v 13.8. The taxonomies were assigned using the RDP classifier to these clusters at ≥97% sequence similarity against the reference Greengenes database. The sequencing stats and absolute read counts are represented in **Tables S2**, **S3**, respectively. PCA and alpha-diversity analysis were performed using MicrobiomeAnalyst. The raw reads data were normalized and relative abundance in % was calculated. For comparative analysis, minimal data filtering was done and features containing non-zero values in less than ~10% samples were removed. Statistical analysis at the levels of phylum, family, and genus was performed by multiple *t*-tests with the assumption that all rows are sample from populations with the same scatter (SD) followed by a two-stage linear step-up procedure of Benjamini, Krieger, and Yekutieli to correct for multiple comparisons by controlling the false discovery rate (<0.05). Due to taxonomic resolution limit of the partial *16S rRNA* gene sequencing-based microbiome study, the abundance difference of the bacterial species in WT and insulin-resistant iNOS^−/−^ mice has not been included in the present study.

### 2.14 Statistical Analysis

Data were presented as mean ± SEM. Independent unpaired Student’s *t*-test was used for comparisons as appropriate using the GraphPad Prism 8 software. More than two groups were compared by one-way analysis of variance (ANOVA) followed by *post-hoc* Tukey’s multiple comparison test or Dunnett’s test. Differences were considered statistically significant at *p <*0.05. For the correlation analysis, Pearson correlation coefficients were calculated and *p*-value was corrected according to the Benjamini–Hochberg correction for multiple comparisons, with a false discovery rate <0.05.

### 2.15 Availability of Data and Materials

All data used in this study are present in the main text and **Supplementary Material**. The raw 16S rRNA gene sequencing data were deposited in the Sequence Read Archive (SRA) of the National Center for Biotechnology Information (NCBI) under accession number PRJNA740126.

## 3 Results

### 3.1 Insulin-Resistant iNOS^−/−^ Mice Display Atypical Gut Microbiota With Gram-Positive Bacteria Dominance and Altered Serum Metabolome

**Figure 1:**
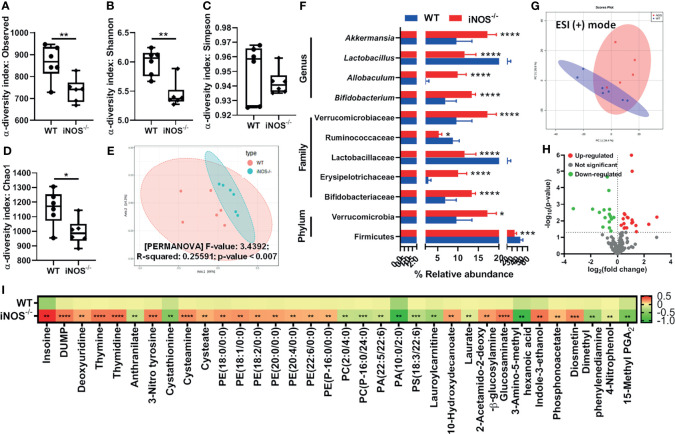
Insulin-resistant iNOS^−/−^ mice display atypical gut microbiota with gram-positive bacteria dominance and altered serum metabolome. Gut microbiota analysis in wild-type (WT) and insulin-resistant iNOS^−/−^ mice. α-Diversity indices in stool samples: **(A)** observed, **(B)** Shannon, **(C)** Simpson, and **(D)** Chao1. **(E)** β-Diversity analysis *via* principal coordinate analysis (PCA) plot based on Bray–Curtis distance. Each dot represents an animal, projected onto the first (horizontal axis) and second (vertical axis) variables. **(F)** Differentially abundant microbiota at the phylum, family, and genus levels. Serum metabolomic analysis in chow-fed WT and iNOS^−/−^ mice in ESI (+) mode. **(G)** PCA score plot and **(H)** volcano plot of differential metabolites (*p* < 0.05) between WT and iNOS^−/−^ mice. Red in the volcano plot indicates significantly upregulated metabolites, green indicates the downregulated metabolites, and gray shows no significant difference. **(I)** Heatmap of differential metabolites found by metabolomics analysis in chow-fed WT and iNOS^−/−^ mice. Data are represented as mean ± SEM (*n* ≥ 6). *p < 0.05, **p < 0.01, ***p < 0.001, ****p < 0.0001 vs. WT. See also **Figure S1**.

Chow-fed iNOS^−/−^ mice were glucose intolerant (**Figures S1A, B**) and systemic insulin resistant (**Figures S1F, G**), hyperglycemic, and hyperinsulinemic as compared with WT (**Figures S1C, D**), along with enhanced HOMA-IR (**Figure S1E**) and decreased QUICKI (**Figure S1H**). iNOS^−/−^ mice also displayed enhanced gluconeogenesis as evident by PTT (**Figures S1K, L**). Circulating total cholesterol, triglycerides (**Figures S1I, J**), LDL, and NEFA were significantly more in iNOS^−/−^ mice, while HDL levels were comparable to WT mice (**Figures S1M**–**O**). The microbial alpha-diversity was reduced in insulin-resistant iNOS^−/−^ mice in the fecal samples as compared with WT (reduced observed, Shannon, and Chao1 diversity indices). However, no significant difference was observed in the Simpson diversity index (**Figures 1A**–**D**). Multidimensional scaling analysis through principal coordinate plot represents that the bacterial communities between the WT and iNOS^−/−^ mice varied significantly (**Figure 1E**). At the phylum level, the relative abundance of Firmicutes was decreased with increased Verrucomicrobia. At the family level, Erysipelotrichaceae, Bifidobacteriaceae, and Verrucomicrobiaceae were increased, while Lactobacillaceae and Ruminococcaceae were decreased. *Allobaculum*, *Bifidobacterium*, and *Akkermansia* were increased significantly, while the *Lactobacillus* genus was reduced in insulin-resistant iNOS^−/−^ mice as compared with WT (**Figure 1F**) indicating differential gut microbiome with major changes in gram-positive bacteria. In the PCA score scatter plot from serum metabolomics, distinctly separated clusters between the WT and iNOS^−/−^ mice in ESI-positive mode were seen (**Figure 1G**) with significantly differential metabolites (*p* < 0.05) being visualized through a volcano plot (**Figure 1H**). iNOS^−/−^ mice displayed enhanced purine and pyridimidine metabolites, PE lipids, PE to PC ratio, 10-hydroxydecanoate, 3-nitrotyrosine, cysteamine, cysteate, carbohydrate metabolites, indole-3-ethanol, diosmetin, and phosphonoacetate. PC, PA, and PS lipids; laurate; lauroyl carnitine; anthranilate; and cystathionine were decreased in iNOS^−/−^ mice suggesting altered metabolic profile as compare with WT (**Figure 1I**).

### 3.2 Vancomycin-Induced Modulation of Gut Microbiota Rescues iNOS^−/−^ Mice From Systemic IR and Dyslipidemia

**Figure 2:**
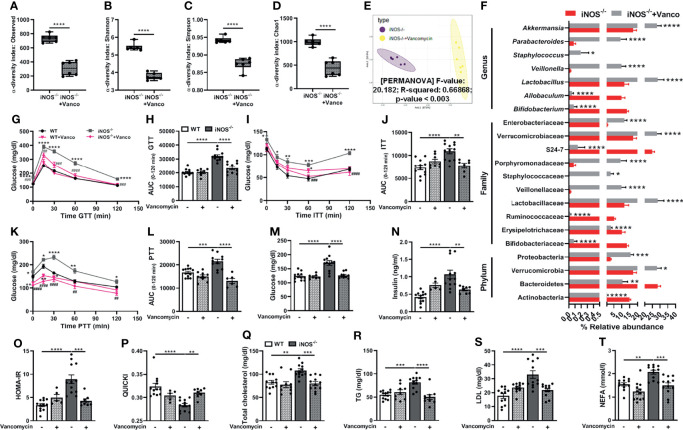
Vancomycin-induced modulation of gut microbiota rescues iNOS^−/−^ mice from systemic IR and dyslipidemia. Gut microbiota analysis in untreated and vancomycin-treated iNOS^−/−^ mice. α-Diversity indices in stool samples: **(A)** observed, **(B)** Shannon, **(C)** Simpson, and **(D)** Chao1. **(E)** PCA plot based on the Bray–Curtis distance. **(F)** Differentially abundant microbiota at the phylum, family, and genus levels. Systemic IR analysis in vancomycin-treated and untreated WT and iNOS^−/−^ mice. **(G)** Intraperitoneal glucose tolerance test (GTT), **(H)** area under the curve (AUC) calculated from IPGTT data, **(I)** intraperitoneal insulin tolerance test (ITT), **(J)** AUC calculated from ITT, **(K)** intraperitoneal pyruvate tolerance test (PTT), **(L)** AUC calculated from PTT, **(M)** fasting blood glucose levels, and **(N)** fasting serum insulin levels. Indices of insulin sensitivity: **(O)** HOMA-IR and **(P)** QUICKI. Serum lipids: **(Q)** total cholesterol (TC), **(R)** triglycerides (TG), **(S)** low-density lipoprotein (LDL), and **(T)** non-esterified free fatty acids (NEFA). Data are represented as mean ± SEM (*n* ≥ 6). *p < 0.05, **p < 0.01, ***p < 0.001, ****p < 0.0001 between indicated groups. ^##^ *p* < 0.01, ^###^ *p* < 0.001 and ^####^ *p* < 0.0001 vs. iNOS^−/−^. See also **Figures S2, S3, S5, S6**.

Subsequently, we used vancomycin to deplete the gram-positive and antibiotic cocktail to deplete both gram-positive and gram-negative gut microbiota in iNOS^−/−^ mice to assess the correlation, if any, between gut microbiome and altered metabolic homeostasis in iNOS^−/−^ mice. Vancomycin (**Figures 2A**–**D**) and Abx (**Figures S3A**–**D**), as expected, further decreased the microbial diversity in insulin-resistant iNOS^−/−^ mice (decreased observed, Shannon, Simpson, and Chao1 α-diversity indices) confirming the depletion of gut microbiota. Abx-treated iNOS^−/−^ mice showed the lowest alpha-diversity among all the groups. Bacterial communities between vancomycin- and Abx-treated WT (**Figures S2E, S5F**) and iNOS^−/−^ mice (**Figures 2E**, **S3E**) vary distinctly and significantly from the untreated controls with markedly different clusters in PCA analysis. At the phylum level, Bacteriodetes and Actinobacteria were decreased significantly in vancomycin-treated WT and iNOS^−/−^ mice with increased Proteobacteria. Verrucomicrobia was increased significantly by vancomycin in iNOS^−/−^ mice. Bacterial families—Bifidobacteriaceae, Ruminococcaceae, S24-7—were decreased significantly in vancomycin-treated WT and iNOS^−/−^ mice with increased Veillonellaceae, Verrucomicrobiaceae, and Enterobacteriaceae. Erysipelotrichaceae was decreased with increased Lactobacillaceae, Staphylococcaceae, and Porphyromonadaceae in vancomycin-treated iNOS^−/−^ mice. Lactobacillaceae and Lachnospiraceae were decreased in vancomycin-treated WT mice. At the genus level, *Bifidobacterium* was decreased with increased *Akkermansia* and *Veillonella* by vancomycin treatment in WT and iNOS^−/−^ mice. Vancomycin decreased *Allobaculum* and increased *Lactobacillus*, *Staphylococcus*, and *Parabacteroides* in iNOS^−/−^ mice, while it decreased *Lactobacillus* and *Oscillospira* in WT (**Figures S2H, 2F**). Abx decreased Firmicutes and Bacteroidetes with increased Proteobacteria in WT and iNOS^−/−^ mice depleting the majority of gut microbiota. Actinobacteria and Verrucomicrobia were decreased by Abx in iNOS^−/−^ mice. Bacterial families—Ruminococcaceae, Lactobacillaceae, and S24-7—were decreased with increased Enterobacteriaceae in Abx-treated WT and iNOS^−/−^ mice. Bifidobacteriaceae, Erysipelotrichaceae, Verrucomicrobiaceae, and Bacteroidaceae were decreased in Abx-treated iNOS^−/−^ mice with increased Brucellaceae in WT. At the genus level, *Lactobacillus*, *Bifidobacterium*, and *Akkermansia* were decreased by Abx treatment in WT and iNOS^−/−^ mice with increased *Klebsiella*. Abx decreased *Allobaculum* and increased *Serratia* in iNOS^−/−^ mice, while it increased *Pseudomonas*, *Elizabethkingia*, and *Ochrobactrum* in WT (**Figures S5H, S3F**). Both vancomycin (**Figures 2G**–**L**) and Abx (**Figures S3G**–**L**) reversed the systemic glucose intolerance and IR and enhanced gluconeogenesis in iNOS^−/−^ mice as seen by a decrease in AUC during GTT, ITT, and PTT, respectively. The increase in the circulating level of glucose and insulin in iNOS^−/−^ mice was decreased by both treatments (**Figures 2M, N**, **S3M, N**). HOMA-IR was decreased by antibiotic treatment with increased QUICKI reversing the IR phenotype observed in iNOS^−/−^ mice (**Figures 2O, P**, **S3O, P**). The enhanced circulating TC, TG, LDL, and NEFA in iNOS^−/−^ mice were reversed by the antibiotic treatment, thus rescuing the dyslipidemia in iNOS^−/−^ mice (**Figures 2Q**–**T**, **S3Q**–**T**). The body weight (**Figures S6A, B**) and food intake (**Figures S6D, E**), however, remained unaltered in obese iNOS^−/−^ mice upon vancomycin and Abx treatment. Body composition and BMI also remained unaltered in vancomycin-treated iNOS^−/−^ mice with increased fat mass in Abx-treated iNOS^−/−^ mice (**Figures S6C, F**). Liver, adipose tissue, and heart weight ratio were decreased in vancomycin- and Abx-treated iNOS^−/−^ mice with increased weight and length ratio of the small intestine and cecum (**Figures S6G, H**).

### 3.3 Vancomycin-Induced Alterations in Serum Metabolites in iNOS^−/−^ Mice

**Figure 3:**
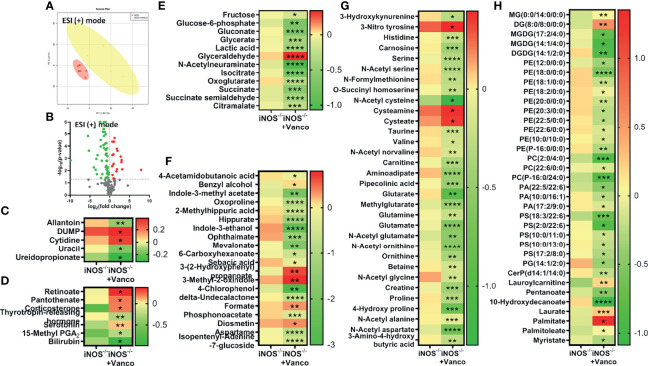
Vancomycin-induced alterations in serum metabolites in iNOS^−/−^ mice. Serum metabolomic analysis in untreated and vancomycin-treated iNOS^−/−^ mice in ESI (+) mode. **(A)** PCA score plot and **(B)** volcano plot of differential metabolites between iNOS^−/−^ mice with or without vancomycin treatment. Red in the volcano plot indicates significantly upregulated metabolites, green indicates the downregulated metabolites, and gray shows no significant difference. Heatmap of differential metabolites found by metabolomics analysis in iNOS^−/−^ mice with and without vancomycin treatment related to **(C)** nucleic acid metabolism; **(D)** vitamins, hormones, and bile acid metabolism; **(E)** carbohydrate metabolism; **(F)** miscellaneous/microbiota-derived metabolites; **(G)** amino acid metabolism; and **(H)** lipid metabolism. Data are represented as mean ± SEM (*n* ≥ 6). *p < 0.05, **p < 0.01, ***p < 0.001, ****p < 0.0001 vs. iNOS^−/−^. See also **Figures S4, S5**.

The PCA score plots of vancomycin (**Figure 3A**) and Abx (**Figure S4A**)-treated and untreated iNOS^−/−^ mice and vancomycin (**Figure S2A**) and Abx (**Figure S5A**)-treated and untreated WT mice in ESI (+) mode showed very distinct separations. Significantly differential metabolites (*p* < 0.05) between untreated iNOS^−/−^ and iNOS^−/−^ treated with vancomycin (**Figure 3B**) or Abx (**Figure S4B**) were visualized through volcano plots. Nucleic acid metabolites—allantoin and pyrimidines—were decreased by vancomycin (**Figure 3C**) and Abx (**Figure S4H**) in iNOS^−/−^ mice with decreased methylthioadenosine by Abx. Vancomycin and Abx treatment did not decrease other purine metabolites in WT and iNOS^−/−^ mice. Methylthioadenosine and allantoin were also decreased in WT by vancomycin (**Figure S2C**) and Abx (**Figure S5D**) along with decreased pyrimidines by Abx. 15-Methyl PGA_2_ and corticosterone were increased by both vancomycin (**Figure 3D**) and Abx (**Figure S4F**) in iNOS^−/−^ mice. Serotonin was increased by vancomycin treatment in iNOS^−/−^ mice with decreased thyrotropin-releasing hormone. The bile acid cholate and the cofactor pyridoxate were decreased by Abx (**Figures S5B, S4F**) in WT and iNOS^−/−^ mice but not by vancomycin (**Figures S2B, 3D**). Glycolysis and Krebs cycle intermediates were decreased in vancomycin (**Figure 3E**) and Abx (**Figure S4D**)-treated iNOS^−/−^ mice, while glyceraldehyde was increased. Vancomycin in WT (**Figure S2F**) did not decrease the carbohydrate metabolites to a significant extent as compared with Abx treatment (**Figure S5C**). Oxoproline, 2-methylhippuric acid, hippurate, indole-3-ethanol, ophthalmate, mevalonate, and aspartame were decreased by vancomycin and Abx in WT (**Figures S2D, S5E**) and iNOS^−/−^ mice (**Figures 3F**, **S4C**). 4-Acetamidobutanoic acid, indole-3-methyl acetate, sebacic acid, 4-chlorophenol, and isopentenyl-adenine-7-glucoside were decreased by vancomycin and Abx in iNOS^−/−^ mice, while formate was increased by vancomycin, suggesting the modulation of bacterial-derived/dependent metabolites by antibiotics. Majority of amino acids were significantly decreased in vancomycin- and Abx-treated WT (**Figures S2G, S5G**) and iNOS^−/−^ mice (**Figures 3G**, **S4E**) including aminoadipate, except 3-nitrotyrosine, cysteamine, and cysteate. The aromatic amino acid metabolite tryptophan was increased in iNOS^−/−^ mice, while 3-methoxytyrosine and N-acetyl phenylalanine were increased in WT by Abx (**Figures S5G, S4E**). Spermidine was increased by vancomycin and Abx in WT mice (**Figures S2G, S5G**). Many lipid species—PE, PC, PA, PS, PG, ceramides, and 10-hydroxydecanoate—were decreased by vancomycin and Abx treatment in iNOS^−/−^ (**Figures 3H**, **S4G**) and WT mice (**Figures S2I, S5I**), while palmitate was increased suggesting improved lipid metabolism along with improvement in PE to PC ratio. Laurate and lauroyl carnitine were decreased in iNOS^−/−^ mice and were increased by vancomycin but not by Abx. These results suggest that vancomycin-induced gram-positive bacteria depletion improved the metabolic perturbations in iNOS^−/−^ mice.

### 3.4 Improvement in Disrupted Lipid and Glucose Homeostasis in the Liver in iNOS^−/−^ Mice Following Treatment With Vancomycin

**Figure 4:**
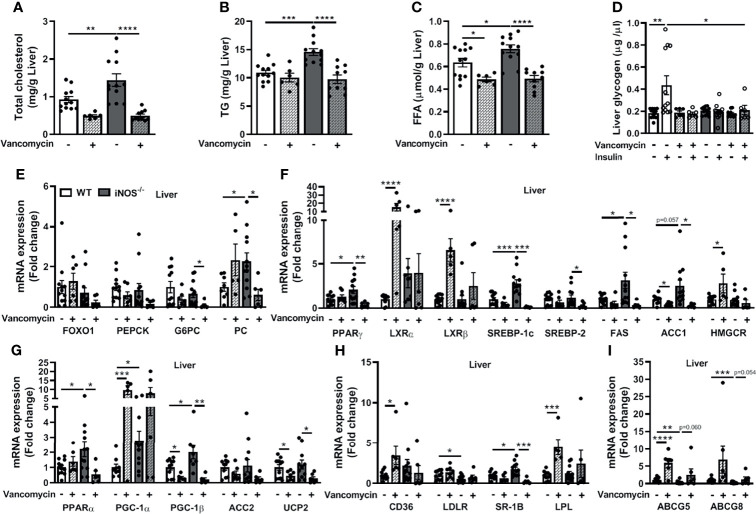
Improvement in the disrupted lipid and glucose homeostasis in the liver in iNOS^−/−^ mice following treatment with vancomycin. Hepatic lipid levels in WT and iNOS^−/−^ mice with and without vancomycin treatment: **(A)** TC, **(B)** TG, and **(C)** FFA. **(D)** Hepatic glycogen levels with or without insulin stimulation in WT and iNOS^−/−^ mice with and without vancomycin treatment. Hepatic mRNA expression analysis of genes involved in **(E)** gluconeogenesis, **(F)** lipid synthesis, **(G)** lipid oxidation, **(H)** lipid uptake, and **(I)** lipid efflux. Data are represented as mean ± SEM (*n* ≥ 6). *p < 0.05, **p < 0.01, ***p < 0.001, ****p < 0.0001 between indicated groups. See also **Figure S7**.

Hepatic TC, TG, and FFA levels were reduced in the vancomycin (**Figures 4A**–**C**) and Abx (**Figures S7A**–**C**)-treated iNOS^−/−^ mice. FFA levels were also decreased in vancomycin- and Abx-treated WT (**Figures 4C**, **S7C**). The enhanced expression of lipid synthesis genes (*PPARγ*, *SREBP-1c*, and *ACC1*) in the liver of iNOS^−/−^ mice was decreased by vancomycin (**Figure 4F**) and Abx (**Figure S7F**) with unchanged *LXRα, LXRβ*, and *HMGCR* in the liver. *FAS* and *SREBP-2* expression was decreased significantly by vancomycin in iNOS^−/−^ mice, but not by Abx. The expression of *LXRα, LXRβ*, and *HMGCR* was increased in the liver of vancomycin-treated WT (**Figure 4F**). PGC-1β expression was decreased in the liver of vancomycin (**Figure 4G**) and Abx (**Figure S7G**)-treated WT and iNOS^−/−^ mice. *PPARα* and *UCP2* expressions were decreased in the liver of Abx-treated WT and vancomycin-treated iNOS^−/−^ mice. Liver *PGC-1α* expression was increased in vancomycin-treated WT. *ACC2* expression remained unchanged in the liver of vancomycin- and Abx-treated iNOS^−/−^ mice. The expression of genes involved in hepatic lipid uptake (*CD36*, *LPL*) was unaltered in iNOS^−/−^ mice following vancomycin (**Figure 4H**) and Abx (**Figure S7H**) treatment with decreased SR-1B. CD36 and LPL expression was increased in vancomycin-treated WT. LDLR expression was reduced in iNOS^−/−^ mice and was unaltered by vancomycin and Abx. The expression of genes involved in hepatic lipid efflux—*ABCG5* and *ABCG8*—was decreased in iNOS^−/−^ mice and was increased significantly in Abx-treated iNOS^−/−^ mice (**Figure S7I**) and were comparable with WT in vancomycin-treated iNOS^−/−^ mice (**Figure 4I**). Hepatic PC expression was decreased by vancomycin with decreased *G6PC* expression by vancomycin (**Figure 4E**) and Abx (**Figure S7E**) in iNOS^−/−^ mice along with unchanged *FOXO1* and *PEPCK*. Hepatic glycogen levels also remained unaltered upon vancomycin treatment (**Figure 4D**), while insulin-stimulated glycogen levels were increased significantly by Abx (**Figure S7D**).

### 3.5 Improvement in the Disrupted Lipid and Glucose Homeostasis in Adipose Tissue and Intestine in iNOS^−/−^ Mice Following Treatment With Vancomycin

**Figure 5:**
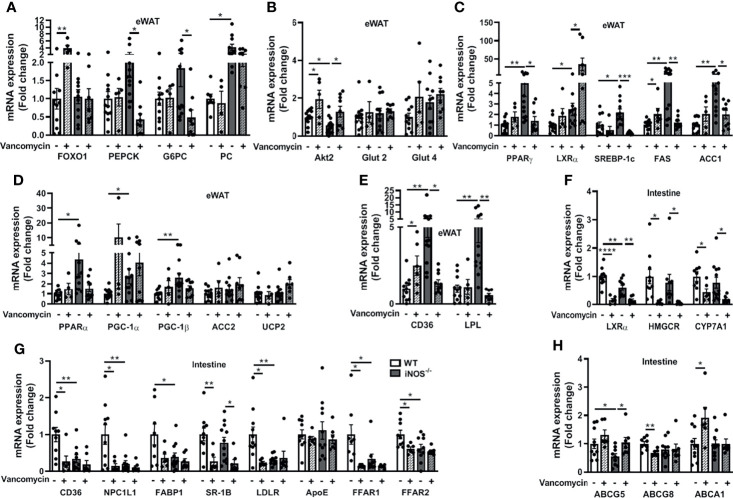
Improvement in the disrupted lipid and glucose homeostasis in adipose tissue and intestine in iNOS^−/−^ mice following treatment with vancomycin. Adipose tissue mRNA expression analysis of genes involved in **(A)** gluconeogenesis, **(B)** glucose homeostasis, **(C)** lipid synthesis, **(D)** lipid oxidation, and **(E)** lipid uptake. Intestinal tissue mRNA expression analysis of genes involved in **(F)** lipid synthesis, **(G)** lipid uptake, and **(H)** lipid efflux. Data are represented as mean ± SEM (*n* ≥ 6). *p < 0.05, **p < 0.01, ***p < 0.001, ****p < 0.0001 between indicated groups. See also **Figures S8, S9**.

The enhanced expressions of lipid synthesis genes (*PPARγ*, *SREBP-1c*, *FAS*, and *ACC1*) in adipose tissue of iNOS^−/−^ mice were decreased by vancomycin, while Abx treatment decreased *SREBP-1c* and *FAS*. *LXRα* expression was increased in the eWAT of vancomycin- (**Figure 5C**) and Abx-treated iNOS^−/−^ mice (**Figure S8C**). *PGC-1α* and *UCP2* expressions in adipose tissue were increased in Abx-treated iNOS^−/−^ mice, while *PGC-1β*, *ACC2*, and *PPARα* remain unchanged by vancomycin (**Figure 5D**) or Abx (**Figure S8D**). The enhanced expression of *CD36* and *LPL* in adipose tissue of iNOS^−/−^ mice was decreased by vancomycin (**Figure 5E**) and Abx treatment (**Figure S8E**). *PEPCK* and *G6PC* were decreased in adipose tissue by vancomycin (**Figure 5A**) and Abx (**Figure S8A**) in iNOS^−/−^ mice with unchanged *FOXO1* and *PC*. Decreased *Akt2* gene expression was increased in white adipose tissue of vancomycin-treated WT and iNOS^−/−^ mice suggesting improved insulin signaling upon gram-positive bacteria depletion. The glucose transporters in adipose tissue remained unaltered upon vancomycin (**Figure 5B**) or Abx treatment (**Figure S8B**).

*LXRα, HMGCR*, and *CYP7A1* expressions were decreased in the small intestine of vancomycin-treated iNOS^−/−^ and WT mice (**Figure 5F**). Abx decreased *LXRα* expression in iNOS^−/−^ and WT mice (**Figure S8F**). The expression of most of the lipid uptake genes in the small intestine (*CD36*, *NPC1L1*, *FABP1*, *LDLR*, *ApoE*, *FFAR1*, *FFAR2*) was not altered in the vancomycin- (**Figure 5G**) and Abx-treated iNOS^−/−^ mice (**Figure S8G**), except *SR-1B*, which was reduced. Furthermore, the expression of *CD36*, *NPC1L1*, *LDLR*, *FFAR1*, and *FFAR2* was reduced in the vancomycin-treated WT mice (**Figure 5G**). *ABCG5* expression was decreased in the small intestine of iNOS^−/−^ mice and was increased by vancomycin (**Figure 5H**) and Abx treatment (**Figure S8H**) with unchanged *ABCG8* and *ABCA1*.

The morphometric calculations (**Figures S9A, D**) displayed increased intestinal villi and colonic crypt length in iNOS^−/−^ mice (**Figures S9C, F**). The barrier functionality of the gut remained unchanged in iNOS^−/−^ mice as assessed by *in-vivo* FITC-dextran gut permeability assay (**Figure S9G**) and Alcian blue staining in intestinal (**Figures S9A, B**) and colonic tissues (**Figures S9D, E**). The gene expression of the tight junction protein *Claudin-2* was increased with decreased *ZO-1* and unaltered *occludin* in iNOS^−/−^ mice. Mucins (*Muc-2* and *Muc-5AC*) remained unaltered in iNOS^−/−^ mice with reduced expression of antimicrobial peptide *Reg3γ* (**Figure S9H**). The FITC-dextran gut permeability assay showed significant reduction in the intestinal permeability following vancomycin and Abx treatment in WT and iNOS^−/−^ mice as compared with untreated controls (**Figure S9G**). The mRNA expression for tight junction proteins, mucins, and antimicrobial peptide in the small intestine largely remained unchanged in antibiotic-treated iNOS^−/−^ mice. *Claudin-2* mRNA expression was increased in Abx-treated WT and iNOS^−/−^mice (**Figure S9H**). Furthermore, the status of NO was checked and the decreased total nitrite levels in iNOS^−/−^ mice were not altered in the serum, adipose tissue, and intestine after treatment with antibiotics. In the liver, the total nitrite levels were further decreased in iNOS^−/−^ mice by antibiotics (**Figures S9I**–**L**). *nNOS* expression remained unchanged in the liver and intestine and was decreased in adipose tissue upon treatment with antibiotics in iNOS^−/−^ mice. Decreased *eNOS* expression was rescued in the adipose tissue and intestine by antibiotics and remained unaltered in the liver (**Figures S9M**–**O**).

### 3.6 Association of Serum Metabolites With Metabolic Profile of iNOS^−/−^ Mice Upon Treatment With Antibiotics

The phenotypic, biochemical, functional, metabolic, and molecular analyses suggest that iNOS^−/−^ mice displayed IR and dyslipidemia along with altered gut microbiome as compared with WT mice. Gut microbiota depletion/modulation by antibiotics showed marked improvement in metabolic parameters with precise alterations in the serum metabolites. Pearson’s correlation was used to identify the serum metabolites which strongly correlated with the metabolic biomarkers quantified in control (WT), insulin-resistant (iNOS^−/−^), and antibiotics (vancomycin or Abx)-treated WT and iNOS^−/−^ mice. The lipid species MGDGs, PEs, PAs, PSs, PGs, ceramides, and 10-hydroxydecanoate positively correlated with bacterial diversity, dyslipidemia, and IR and negatively with cecum weight. Laurate and palmitate were negatively correlated liver lipids and also negatively correlated with bacterial diversity (**Figure S10A**). Majority of amino acids positively correlated with biomarkers of IR, dyslipidemia, and α-diversity and negatively correlated with HDL levels and cecum weight (**Figure S10B**).

Indole-3-methyl acetate, 5-oxoproline, 2-methylhippuric acid, hippurate, indole-3-ethanol, ophthalmate, mevalonate, 6-carboxyhexanoate, sebacic acid, 4-chlorophenol, δ-undecalactone, and aspartame positively correlated with IR, dyslipidemia, and α-diversity and negatively correlated with cecum weight. S-carboxymethylcysteine and formate were negatively correlated with dyslipidemia and α-diversity (**Figure S10C**). Nucleic acid metabolites—methylthioadenosine, xanthine, allantoin, thymine, cytosine, and uracil—positively correlated with biomarkers of IR and dyslipidemia and negatively correlated with cecum weight. Glycolysis and Krebs cycle intermediates were correlated positively and glyceraldehyde negatively with liver lipids and α-diversity. 4-Pyridoxate and melatonin were positively correlated with glucose intolerance and dyslipidemia. Bile acids and bile pigments were positively correlated with IR and dyslipidemia and negatively correlated with cecum weight (**Figure S10D**). These results suggest that serum metabolites exhibited strong association with specific metabolic biomarkers and were modulated by microbiota depletion by antibiotics.

### 3.7 Association of Gut Microbiota With Metabolic Parameters in iNOS^−/−^ Mice Following Treatment With Antibiotics

**Figure 6:**
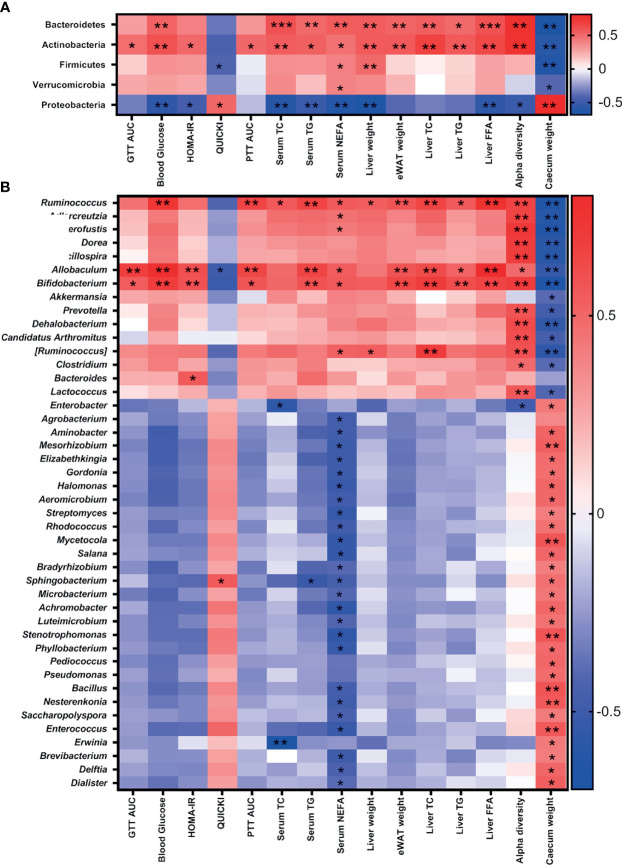
Association of gut microbiota with metabolic parameters in iNOS^−/−^ mice following treatment with antibiotics. Heatmap of Pearson’s correlation coefficients between changes in different metabolic parameters and microbial taxa at the **(A)** phylum and **(B)** genus levels caused by gut microbiota modulation by vancomycin and Abx in WT and iNOS^−/−^ mice. *p < 0.05, **p < 0.01, ***p < 0.001 represent significant correlations between metabolic biomarker and bacterial taxa. Blue color represents negative and red positive correlations. See also **Figure S11**.

Furthermore, the association between antibiotic-induced compositional changes in microbiota with the alterations in metabolic parameters was investigated. The phyla Bacteroidetes and Actinobacteria were positively associated with IR, dyslipidemia, and α-diversity, while Proteobacteria correlated negatively. Firmicutes and Verrucomicrobia were negatively correlated with cecum weight and positively correlated with dyslipidemia (**Figure 6A**). Bifidobacteriaceae (Actinobacteria), Erysipelotrichaceae (Firmicutes), and S24-7 (Bacteroidetes) were positively correlated with IR, dyslipidemia, and α-diversity and negatively with cecum weight. The microbial families—Ruminococcaceae, Eubacteriaceae, Lachnospiraceae, Dehalobacteriaceae, Mogibacteriaceae, and Lactobacillaceae (Firmicutes); Coriobacteriaceae (Actinobacteria); and Verrucomicrobiaceae (Verrucomicrobia)—were positively correlated with dyslipidemia and α-diversity and negatively correlated with cecum weight. Weeksellaceae and Sphingobacteriaceae (Bacteroidetes); Enterococcaceae, Bacillaceae, Tissierellaceae, and Leuconostocaceae (Firmicutes); Microbacteriaceae, Micrococcaceae, Gordoniaceae, Nocardiaceae, Nocardiodaceae, Promicromonosporaceae, Beutenbergiaceae, Streptomycetaceae, Pseudonocardiaceae, Brevibacteriaceae, and Geodermatophilaceae (Actinobacteria); and many families of Proteobacteria including Enterobacteriaceae were negatively correlated with serum NEFA and positively correlated with cecum weight (**Figure S11**).

*Bifidobacterium* (Actinobacteria) and *Allobaculum* and *Ruminococcus* (Firmicutes) were positively correlated with IR, dyslipidemia, and α-diversity and negatively with cecum weight. *Anaerofustis* (Firmicutes) and *Adlercreutzia* (Actinobacteria) were positively correlated with serum NEFA and α-diversity and negatively correlated with cecum weight. *Pediococcus*, *Bacillus*, *Enterococcus*, and *Dialister* (Firmicutes); *Sphingobacterium* and *Elizabethkingia* (Bacteroidetes); *Gordonia*, *Aeromicrobium*, *Streptomyces*, *Rhodococcus*, *Mycetocola*, *Salana*, *Microbacterium*, *Luteimicrobium*, *Nesterkonia*, *Saccharopolyspora*, and *Brevibacterium* (Actinobacteria); and many genus of Proteobacteria phylum including *Enterobacter* were negatively correlated with NEFA and positively correlated with cecum weight (**Figure 6B**). These results suggest that gram-positive bacteria depletion (*Allobaculum*, *Bifidobacterium*, and *Ruminococcus*) following treatment with vancomycin is directly correlated with the improvement in metabolic biomarkers.

### 3.8 Gut Microbiota Association With Serum Metabolites in iNOS^−/−^ Mice Upon Antibiotics Treatment

Pearson’s correlation coefficients were calculated between serum metabolites and microbial taxa, which positively correlated with blood glucose and glucose intolerance in WT and iNOS^−/−^ mice without or with antibiotics (vancomycin or Abx) treatment to gain better insights into the host metabolism. Interestingly, most of these microbial taxa linked to impaired glucose metabolism are gram-positive bacteria. The lipid species MGDG, PE, PC, PA, PS, PG, ceramide, 10-hydroxydecanoate, dodecanedioic acid, and myristate were positively correlated and palmitate was negatively correlated with taxa directly associated with glucose intolerance (Actinobacteria, Bacteroidetes, Erysipelotrichaceae, Bifidobacteriaceae, S24-7, Ruminococcaceae, *Allobaculum*, *Bifidobacterium*, *Ruminococcus*) (**Figure S12A**). Most amino acid metabolites including anthranilate, histidine, carnosine, serine, N-formylmethionine, N-acetyl cysteine, N-acetyl norvaline, carnitine, aminoadipate, pipecolinic acid, methyl glutarate, glutamate, N-acetyl ornithine, betaine, creatine, creatinine, proline, leucyl proline, and 3-amino-4-hydroxybutyric acid were positively correlated with taxa associated with glucose intolerance (**Figure S12B**).

The metabolites indole-3-methyl acetate, 5-oxoproline, 2-methylhippuric acid, hippurate, indole-3-ethanol, ophthalmate, mevalonate, 6-carboxyhexanoate, sebacic acid, 4-chlorophenol, δ-undecalactone, aspartame, and bis(2-ethylhexyl)phthalate were positively correlated and S-carboxymethylcysteine and formate were negatively correlated with taxa associated with glucose intolerance (**Figure S12C**). Purines (allantoin, methylthioadenosine) and pyrimidines (uracil) correlated positively with taxa associated with glucose intolerance. Lactic acid, N-acetyl neuraminate, isocitrate, and citramalate were positively correlated, while glyceraldehyde was negatively correlated with taxa associated with glucose intolerance. 4-Pyridoxate, melatonin, bile acids, and pigments (cholate, deoxycholate, and bilirubin) were positively correlated with taxa associated with glucose intolerance (**Figure S12D**). These results suggest that metabolites involved in lipids, amino acids, bile acids, nucleic acids, carbohydrate, and bacterial-derived/dependent metabolism exhibited significant correlation with gram-positive bacterial taxa linked to IR and dyslipidemia in iNOS^−/−^ mice.

## 4 Discussion

Involvement of both gram-positive and gram-negative bacteria has been reported in diabetes and metabolic syndrome; however, most of the studies on gram-negative bacteria linked LPS-mediated inflammation with metabolic dysregulation (Rawat et al., 2012; Pushpanathan et al., 2016; Radilla-Vázquez et al., 2016; Liu et al., 2019). iNOS^−/−^ mice are, however, protected against LPS-induced inflammation and the associated metabolic perturbations (Chauhan et al., 2003; Carvalho-Filho et al., 2006; House et al., 2012). iNOS^−/−^ mice were insulin resistant and dyslipidemic on chow, LFD, and HFD diets, which was improved substantially by nitrite supplementation (Kanuri et al., 2017; Aggarwal et al., 2019; Pathak et al., 2019; Aggarwal et al., 2020). To investigate further the role of gut bacteria in metabolic perturbations in iNOS^−/−^ mice, we checked the microbial diversity in fecal samples. Reduced bacterial diversity in iNOS^−/−^ mice correlated with less diverse microbial communities in obese subjects (Turnbaugh et al., 2009). Similar to that reported in ileum (Matziouridou et al., 2017), we observed increased relative abundance of *Bifidobacterium* and *Allobaculum* in the feces belonging to the phyla Actinobacteria and Firmicutes, respectively, in iNOS^−/−^ mice. Actinobacteria are augmented in obese subjects and in patients with liver diseases and diabetes (Turnbaugh et al., 2009). *Allobaculum*, the efficient energy harvesters and active assimilators of glucose (Herrmann et al., 2017), was enhanced in LFD- and HFD-fed mice (Ravussin et al., 2012), during metabolic dysbiosis (Nobel et al., 2015), and in diabetic ZDF rats (Zhou et al., 2019). *Allobaculum* is a secondary degrader and seems to be dependent on primary degraders like *Bifidobacterium* for the utilization of complex polysaccharides for their growth. We and others (Kim et al., 2017; Zhou et al., 2019) did find a positive correlation between *Bifidobacterium*, *Ruminococcus*, and *Allobaculum* in the hyperglycemic condition. Gut barrier dysfunction and increased availability of microbial products in the systemic circulation, commonly known as metabolic endotoxemia, are considered as key drivers of obesity and metabolic syndrome (Cani et al., 2007). Surprisingly, iNOS-deficient mice did not display alterations in gut permeability, possibly due to an increase in the relative abundance of *Akkermansia muciniphila*, a gram-negative bacteria that is shown to maintain and restore mucosal barrier integrity in obese and diabetic mice and in inflammatory conditions (Everard et al., 2013; Bian et al., 2019) and is reported to be a beneficial microbe in maintaining metabolic health. However, despite its beneficial effects, we observed metabolic disruptions in iNOS^−/−^ mice. Intestinal villi and colon crypt length were also increased in iNOS^−/−^ mice, and this could be an adaptation for improved absorption of nutrients under hyperglycemic conditions (Isah and Masola, 2017).

As iNOS^−/−^ mice exhibited a higher abundance of the gram-positive bacteria *Allobaculum* and *Bifidobacterium*, we employed vancomycin, which largely depletes gram-positive bacteria to study their association with IR and dyslipidemia. The vancomycin intervention altered the gut microbiome profiles as reported earlier (Tulstrup et al., 2015; Ray et al., 2021) in both WT and iNOS^−/−^ mice with decreased bacterial alpha-diversity and increased relative abundance of Proteobacteria (Ray et al., 2021), the signature markers of antibiotic treatment. Proteobacteria exhibit metabolic plasticity (Stecher et al., 2012) and are reservoirs of several horizontally acquired antimicrobial resistance genes (Jiang et al., 2017) leading to their expansion after treatment with antibiotics. Intriguingly, vancomycin-induced reduction of *Allobaculum*, *Bifidobacterium*, and *Ruminococcus* was linked with improved IR and dyslipidemia in iNOS ^−/−^ mice. Antibiotics strengthen the intestinal integrity (Tulstrup et al., 2015; Fujisaka et al., 2016) with no distinguishable change in mucins and tight junction proteins (Tulstrup et al., 2015; Ray et al., 2021) as was also observed in iNOS^−/−^ mice. The depletion of microbiota by treatment with antibiotics in WT (Rodrigues et al., 2017; Kuno et al., 2018; Zarrinpar et al., 2018) and in diet-induced obese mice (Cani et al., 2008; Membrez et al., 2008; Carvalho et al., 2012; Hwang et al., 2015) improved glucose tolerance and IR as found by us in iNOS^−/−^ mice. *Veillonella* has recently been reported to improve cardiovascular health and exercise performance by utilizing lactic acid and converting it into propionate (Scheiman et al., 2019) which might have a symbiotic relationship with the lactic acid-producing *Lactobacillus.* This might have been the reason for the expansion of both *Veillonella* and *Lactobacillus* in vancomycin-treated iNOS^−/−^ mice. The susceptibility of *Lactobacilli* to vancomycin varies as *Lactobacillus acidophilus* and *Lact. delbreuckii* are vancomycin sensitive, while *Lact. rhamnosus* are vancomycin resistant (Hamilton-Miller and Shah, 1998). We also used a broad-spectrum antibiotic cocktail to deplete the majority of gut gram-positive and gram-negative bacteria so as to further assess the role of gut microbiota in iNOS^−/−^ mice. As expected, *Allobaculum*, *Bifidobacterium*, *Lactobacillus*, and *Akkermansia* were also depleted by Abx, with metabolic improvements along with reversal of IR and dyslipidemia. *Lactobacillus rhamnosus* and *Lactobacillus casei* improved glucose tolerance and IR in db/db mice (Park et al., 2015), HFD-fed obese mice (Kim et al., 2013), and diabetic mice (Li et al., 2017b); however, Abx treatment in this study also reduced *Lactobacillus*. We therefore failed to notice any additional advantage in IR and metabolism. Captivatingly, vancomycin and the antibiotic cocktail reshaped the gut microbiome and the correlation analysis suggests the importance of gram-positive bacteria *Allobaculum*, *Bifidobacterium*, and *Ruminococcus* depletion in the reversal of IR and dyslipidemia in iNOS^−/−^ mice with a seemingly less important role of gram-negative bacteria in the redox imbalanced state.

Reduced gluconeogenesis was evident by the change in PTT and a decrease in the expression of gluconeogenic enzymes in the liver and adipose tissue of iNOS^−/−^ mice after treatment with antibiotics (Membrez et al., 2008; Zarrinpar et al., 2018). A decrease in the insulin-stimulated glycogen levels in iNOS^−/−^ mice suggests enhanced flux of glucose into lipogenic pathways and their storage (Irimia et al., 2017), which was enhanced by Abx as reported previously in ob/ob mice (Membrez et al., 2008). Treatment with antibiotics improved insulin signaling in iNOS^−/−^ mice *via* Akt (Carvalho et al., 2012). Interestingly, treatment with antibiotics rescued dyslipidemia (Kuno et al., 2018) in iNOS^−/−^ mice *via* reduction in the expression of genes involved in fatty acid synthesis (Zarrinpar et al., 2018) in the liver and adipose tissue and of lipid uptake in the adipose tissue, while the expression of genes involved in the lipid efflux was augmented in the liver and intestine.

Systemic changes in metabolism were reflected by untargeted metabolomics in the serum and revealed that purine and pyrimidine metabolites were elevated in iNOS^−/−^ mice as supported by other studies in diabetic human subjects and rodents (Dudzinska, 2014; Urasaki et al., 2016). Bile acids and bile salts have been positively correlated with IR in humans (Cariou et al., 2011) and also in iNOS^−/−^ mice and were decreased upon gut microbiota depletion by antibiotics as reported previously in HFD-fed mice (Fujisaka et al., 2016; Zarrinpar et al., 2018). Lipids are the major source of energy metabolism and are independent predictors for impairment of insulin actions and progression to diabetes (Zhao et al., 2017). The present study revealed that lipid metabolism seems to be the principal disordered pathway in iNOS^−/−^ mice. Following vancomycin treatment, dyslipidemia and IR were reversed suggesting an association of lipid species with glucose intolerance. It has been reported that in NAFLD/NASH in humans, PE concentration is increased relative to the disease progression (Ma et al., 2016) and NASH patients have a decreased ratio of PC to PE (Li et al., 2006). Nozaki et al. observed higher propensity of fatty liver damage in iNOS^−/−^ mice (Nozaki et al., 2015), and perturbed PE/PC ratio observed by us in iNOS^−/−^ mice might provide a plausible explanation. Moreover, the PE/PC ratio was improved upon treatment with antibiotics in iNOS^−/−^ mice similar to WT along with metabolic improvements by gram-positive bacteria depletion. Hydroxydecanoate is enhanced in obesity-associated IR and type 2 diabetes (Al-Sulaiti et al., 2019); we, similarly, observed an increase in this metabolite in iNOS^−/−^ mice and a reduction with vancomycin intervention. Laurate and lauroyl carnitine were decreased in iNOS^−/−^ mice, which could be due to the enhanced oxidation of fatty acids, as they are suggested to be accumulated during defects in fatty acid oxidation in diabetic states (Möder et al., 2003; Nowak et al., 2018). Interestingly, laurate and lauroyl carnitine were increased upon vancomycin treatment specifically and not by antibiotic cocktail in iNOS^−/−^ mice suggesting the role of vancomycin-selective gram-positive bacteria in their metabolism.

Hippurate, a bacterial metabolite, is an early biomarker of IR and diabetes (Zhang, 2015). Allantoin, an oxidative stress biomarker, is the end product of uric acid oxidation, and it is enhanced in streptozotocin-induced diabetic rats, db/db mice, obese dogs, and gestational diabetic females (Hernandez-Baixauli et al., 2020). Gram-positive bacteria are positive contributors of hippurate, allantoin, glucose, 2-oxoglutarate, and glutamine as these were found to be present in the serum of mice infected with gram-positive bacteria (Hoerr et al., 2012). In the present study following vancomycin-induced depletion of gram-positive bacteria, these metabolites were reduced in iNOS^−/−^ mice. Formate, produced by gram-negative bacteria (Hoerr et al., 2012), was increased in vancomycin-treated iNOS^−/−^ mice but not by antibiotic cocktail treatment, suggesting the differential metabolic response of gram-positive and gram -negative bacteria. Indole-3-ethanol, a bacterial-derived metabolite of tryptophan (Roager and Licht, 2018), was increased in iNOS^−/−^ mice. Sebacic acid was higher in diabetic (Hameed et al., 2020) and NAFLD patients (Liu et al., 2021). Aminoadipate, a diabetogen, is reported to be involved in glycemic perturbations (Wang et al., 2013). Ophthalmate is a potential biomarker for oxidative stress and glutathione depletion (Mehta et al., 2019). These bacteria-derived metabolites were decreased upon microbiota depletion by antibiotics, suggesting their altered metabolism and possible association with IR in iNOS^−/−^ mice. The present study demonstrates an association of the metabolic perturbations in iNOS^−/−^ mice with select gut microbiota and serum metabolites. It might be helpful if the causative effect of these gut bacteria and metabolites could be also confirmed by implanting these bacteria in germ-free and conventionally raised iNOS^−/−^ and WT mice. The dominance of gram-positive bacteria in insulin-resistant iNOS^−/−^ mice and the improvement in metabolic markers with vancomycin intervention indicate that gram-positive bacteria are crucial drivers of observed metabolic phenotype in these mice. However, we do not completely rule out the role of gram-negative bacteria and other microorganisms such as archaea in regulating the metabolic functions. The association of the gram-negative bacteria, if any, could be also examined in iNOS^−/−^ mice as this could not be investigated in our study. Understanding the interplay between nutrition, genetics, and microbial metabolism with redox imbalance is necessary to combat the scourge of IR.

**Figure 7:**
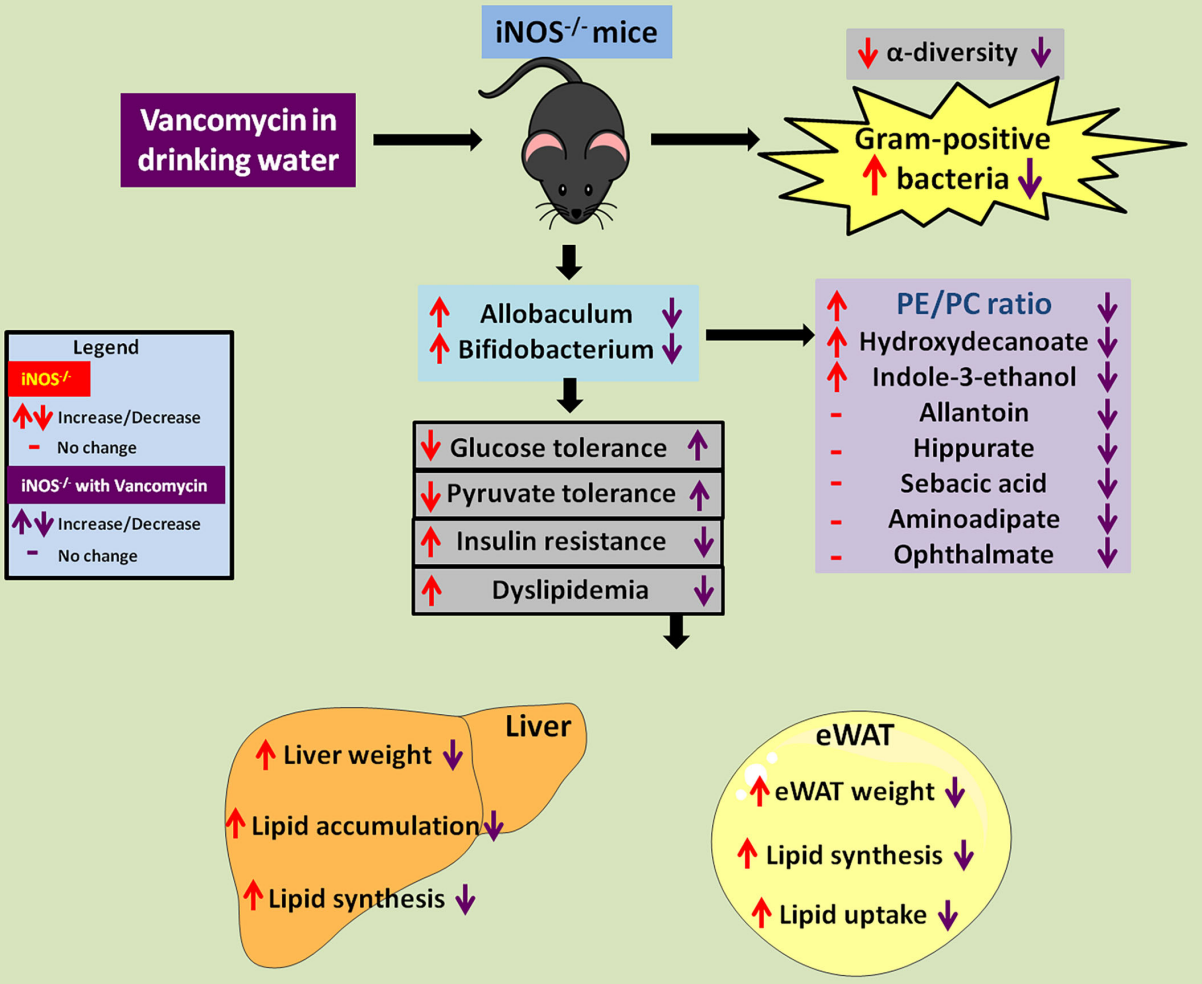
Vancomycin-induced depletion of gram-positive gut bacteria and modulation of associated metabolites rescue the IR and dyslipidemia observed in iNOS^−/−^ mice. Red up or down arrows or dash represents increase, decrease, or no change in iNOS^−/−^ mice in comparison with WT. Violet up or down arrows or dash represents increase, decrease, or no change in vancomycin-treated iNOS^−/−^ mice respectively in comparison with untreated iNOS^−/−^.

Altogether, in the present study, we report that increased relative abundance of gram-positive *Allobaculum* and *Bifidobacterium* in redox imbalanced iNOS^−/−^ mice led to metabolic perturbations with enhanced PE/PC ratio, 10-hydroxydecanate, and indole-3-ethanol levels. Vancomycin-mediated depletion of *Allobaculum*, *Bifidobacterium*, and *Ruminococcus* was associated with the reversal of IR and dyslipidemia in iNOS^−/−^ mice. Vancomycin treatment also led to improvement in PE/PC ratio, 10-hydroxydecanoate, indole-3-ethanol, allantoin, hippurate, sebacic acid, aminoadipate, and ophthalmate (**Figure 7**). Overall, the results obtained in the present study demonstrate that gram-positive gut microbiota, the associated metabolites, and their crosstalk with the host drive dyslipidemia and insulin resistance in iNOS^−/−^ mice.

## Data Availability Statement

The datasets presented in this study can be found in online repositories. The names of the repository/repositories and accession number(s) can be found in the article/**Supplementary Material**.

## Ethics Statement

The animal study was reviewed and approved by the Institutional Animal Ethics Committee of CSIR-CDRI (IAEC/2014/43) in accordance with CPCSEA guidelines.

## Author Contributions

HA performed most of the experiments and wrote the manuscript. PP performed some experiments. VS provided deeper understanding and critical suggestions during the study and manuscript finalization. MS provided critical inputs and interpretation for gut microbiome studies/data. YK performed the metabolomics experiments and analyzed the data. BD provided critical inputs regarding fecal DNA isolation and interpretation for gut microbiome studies/data. KJ provided the animals and critical suggestions during the study. MD conceptualized the whole project, designed and supervised the studies, and interpreted the data as being presented in the MS; she also edited and finalized the manuscript. All authors contributed to the article and approved the submitted version.

## Funding

The present study was supported by JC Bose National fellowship (SB/SE/JCB-017/2015) and THSTI core grant to MD and by DST SERB Project (EMR/2017/003730) to KJ (CSIR-CDRI). Research fellowships to HA from the Indian Council of Medical Research and to PP from the Council of Scientific and Industrial Research, India, are acknowledged.

## Conflict of Interest

The authors declare that the research was conducted in the absence of any commercial or financial relationships that could be construed as a potential conflict of interest.

## Publisher’s Note

All claims expressed in this article are solely those of the authors and do not necessarily represent those of their affiliated organizations, or those of the publisher, the editors and the reviewers. Any product that may be evaluated in this article, or claim that may be made by its manufacturer, is not guaranteed or endorsed by the publisher.

## References

[B1] Aggarwal H. Kanuri B. N. Dikshit M. (2019). “Role of iNOS in Insulin Resistance and Endothelial Dysfunction,” in Oxidative Stress in Heart Diseases (Springer Singapore: Singapore), 461–482. doi: 10.1007/978-981-13-8273-4_21

[B2] Aggarwal H. Pathak P. Kumar Y. Jagavelu K. Dikshit M. (2022). Modulation of Insulin Resistance, Dyslipidemia and Serum Metabolome in iNOS Knockout Mice Following Treatment With Nitrite, Metformin, Pioglitazone, and a Combination of Ampicillin and Neomycin. Int. J. Mol. Sci 23 (1), 195. doi: 10.3390/ijms23010195 PMC8745663 35008623

[B3] Aggarwal H. Pathak P. Singh P. Gayen J. R. Jagavelu K. Dikshit M. (2020). Systemic Insulin Resistance and Metabolic Perturbations in Chow Fed Inducible Nitric Oxide Synthase Knockout Male Mice: Partial Reversal by Nitrite Supplementation. Antioxidants 9, 736. doi: 10.3390/antiox9080736 PMC7465804 32806494

[B4] Ahlqvist E. Storm P. Käräjämäki A. Martinell M. Dorkhan M. Carlsson A.. (2018). Novel Subgroups of Adult-Onset Diabetes and Their Association With Outcomes: A Data-Driven Cluster Analysis of Six Variables. Lancet Diabetes Endocrinol. 6, 361–369. doi: 10.1016/S2213-8587(18)30051-2 29503172

[B5] Al-Sulaiti H. Diboun I. Agha M. V. Mohamed F. F. S. Atkin S. Dömling A. S.. (2019). Metabolic Signature of Obesity-Associated Insulin Resistance and Type 2 Diabetes. J. Transl. Med. 17, 348. doi: 10.1186/S12967-019-2096-8 31640727 PMC6805293

[B6] Bag S. Saha B. Mehta O. Anbumani D. Kumar N. Dayal M.. (2016). An Improved Method for High Quality Metagenomics DNA Extraction From Human and Environmental Samples. Sci. Rep. 6, 26775. doi: 10.1038/srep26775 27240745 PMC4886217

[B7] Bian X. Wu W. Yang L. Lv L. Wang Q. Li Y.. (2019). Administration of Akkermansia Muciniphila Ameliorates Dextran Sulfate Sodium-Induced Ulcerative Colitis in Mice. Front. Microbiol. 10, 2259. doi: 10.3389/fmicb.2019.02259 31632373 PMC6779789

[B8] Bogdan C. (2015). Nitric Oxide Synthase in Innate and Adaptive Immunity: An Update. Trends Immunol. 36, 161–178. doi: 10.1016/j.it.2015.01.003 25687683

[B9] Cani P. D. Amar J. Iglesias M. A. Poggi M. Knauf C. Bastelica D.. (2007). Metabolic Endotoxemia Initiates Obesity and Insulin Resistance. Diabetes 56, 1761–1772. doi: 10.2337/DB06-1491 17456850

[B10] Cani P. D. Bibiloni R. Knauf C. Waget A. Neyrinck A. M. Delzenne N. M.. (2008). Changes in Gut Microbiota Control Metabolic Endotoxemia-Induced Inflammation in High-Fat Diet-Induced Obesity and Diabetes in Mice. Diabetes 57, 1470–1481. doi: 10.2337/db07-1403 18305141

[B11] Cariou B. Chetiveaux M. Zar Y. Pouteau E. Disse E. Guyomarc’H-Delasalle B.. (2011). Fasting Plasma Chenodeoxycholic Acid and Cholic Acid Concentrations Are Inversely Correlated With Insulin Sensitivity in Adults. Nutr. Metab. 8, 48. doi: 10.1186/1743-7075-8-48 PMC3143920 21736725

[B12] Carvalho-Filho M. A. Ueno M. Carvalheira J. B. C. Velloso L. A. Saad M. J. A. (2006). Targeted Disruption of iNOS Prevents LPS-Induced *S* -Nitrosation of Irβ/IRS-1 and Akt and Insulin Resistance in Muscle of Mice. Am. J. Physiol. Metab. 291, E476–E482. doi: 10.1152/ajpendo.00422.2005 16638822

[B13] Carvalho B. M. Guadagnini D. Tsukumo D. M. L. Schenka A. A. Latuf-Filho P. Vassallo J.. (2012). Modulation of Gut Microbiota by Antibiotics Improves Insulin Signalling in High-Fat Fed Mice. Diabetologia 55, 2823–2834. doi: 10.1007/s00125-012-2648-4 22828956

[B14] Cha H.-N. Song S. E. Kim Y.-W. Kim J.-Y. Won K.-C. Park S.-Y. (2011). Lack of Inducible Nitric Oxide Synthase Prevents Lipid-Induced Skeletal Muscle Insulin Resistance Without Attenuating Cytokine Level. J. Pharmacol. Sci. 117, 77–86. doi: 10.1254/jphs.11093FP 22001626

[B15] Chauhan S. D. Seggara G. Vo P. A. Macallister R. J. Hobbs A. J. Ahluwalia A. (2003). Protection Against Lipopolysaccharide-Induced Endothelial Dysfunction in Resistance and Conduit Vasculature of iNOS Knockout Mice. FASEB J. 17, 773–775. doi: 10.1096/FJ.02-0668FJE 12586741

[B16] Cox L. M. Blaser M. J. (2013). Pathways in Microbe-Induced Obesity. Cell Metab. 17, 883–894. doi: 10.1016/j.cmet.2013.05.004 23747247 PMC3727904

[B17] Cui J. Le G. Yang R. Shi Y. (2009). Lipoic Acid Attenuates High Fat Diet-Induced Chronic Oxidative Stress and Immunosuppression in Mice Jejunum: A Microarray Analysis. Cell. Immunol. 260, 44–50. doi: 10.1016/j.cellimm.2009.08.001 19766202

[B18] Dethlefsen L. Huse S. Sogin M. L. Relman D. A. (2008). The Pervasive Effects of an Antibiotic on the Human Gut Microbiota, as Revealed by Deep 16s rRNA Sequencing. PloS Biol. 6, 2383–2400. doi: 10.1371/journal.pbio.0060280 PMC2586385 19018661

[B19] Dudzinska W. (2014). Purine Nucleotides and Their Metabolites in Patients With Type 1 and 2 Diabetes Mellitus. J. Biomed. Sci. Eng. 07, 38–44. doi: 10.4236/jbise.2014.71006

[B20] Eid H. M. Wright M. L. Anil Kumar N. V. Qawasmeh A. Hassan S. T. S. Mocan A.. (2017). Significance of Microbiota in Obesity and Metabolic Diseases and the Modulatory Potential by Medicinal Plant and Food Ingredients. Front. Pharmacol. 0, 387. doi: 10.3389/FPHAR.2017.00387 PMC5493053 28713266

[B21] Everard A. Belzer C. Geurts L. Ouwerkerk J. P. Druart C. Bindels L. B.. (2013). Cross-Talk Between Akkermansia Muciniphila and Intestinal Epithelium Controls Diet-Induced Obesity. Proc. Natl. Acad. Sci. U. S. A. 110, 9066–9071. doi: 10.1073/PNAS.1219451110 23671105 PMC3670398

[B22] Fujisaka S. Ussar S. Clish C. Devkota S. Dreyfuss J. M. Sakaguchi M.. (2016). Antibiotic Effects on Gut Microbiota and Metabolism Are Host Dependent. J. Clin. Invest 126, 4430–4443. doi: 10.1172/JCI86674 27775551 PMC5127688

[B23] Hameed A. Mojsak P. Buczynska A. Suleria H. A. R. Kretowski A. Ciborowski M. (2020). Altered Metabolome of Lipids and Amino Acids Species: A Source of Early Signature Biomarkers of T2DM. J. Clin. Med. 9, 2257. doi: 10.3390/jcm9072257 PMC7409008 32708684

[B24] Hamilton-Miller J. M. T. Shah S. (1998). Vancomycin Susceptibility as an Aid to the Identification of Lactobacilli. Lett. Appl. Microbiol. 26, 153–154. doi: 10.1046/j.1472-765X.1998.00297.x 9569701

[B25] Hansen C. H. F. Krych L. Nielsen D. S. Vogensen F. K. Hansen L. H. Sørensen S. J.. (2012). Early Life Treatment With Vancomycin Propagates Akkermansia Muciniphila and Reduces Diabetes Incidence in the NOD Mouse. Diabetologia 55, 2285–2294. doi: 10.1007/s00125-012-2564-7 22572803

[B26] Hernandez-Baixauli J. Quesada-Vázquez S. Mariné-Casadó R. Cardoso K. G. Caimari A. Del Bas J. M.. (2020). Detection of Early Disease Risk Factors Associated With Metabolic Syndrome: A New Era With the NMR Metabolomics Assessment. Nutr. 12, 806. doi: 10.3390/NU12030806 PMC7146483 32197513

[B27] Herrmann E. Young W. Rosendale D. Reichert-Grimm V. Riedel C. U. Conrad R.. (2017). RNA-Based Stable Isotope Probing Suggests Allobaculum Spp. As Particularly Active Glucose Assimilators in a Complex Murine Microbiota Cultured *In Vitro* . BioMed Res. Int. doi: 10.1155/2017/1829685 PMC5337319 28299315

[B28] Hoerr V. Zbytnuik L. Leger C. Tam P. P. C. Kubes P. Vogel H. J. (2012). Gram-Negative and Gram-Positive Bacterial Infections Give Rise to a Different Metabolic Response in a Mouse Model. J. Proteome Res. 11, 3231–3245. doi: 10.1021/PR201274R 22483232 PMC3368387

[B29] Hollenberg S. M. Broussard M. Osman J. Parrillo J. E. (2000). Increased Microvascular Reactivity and Improved Mortality in Septic Mice Lacking Inducible Nitric Oxide Synthase. Circ. Res. 86, 774–779. doi: 10.1161/01.res.86.7.774 10764411

[B30] House Ii L. M. Morris R. T. Barnes T. M. Lantier L. Cyphert T. J. Mcguinness O. P.. (2012). Tissue Inflammation and Nitric Oxide-Mediated Alterations in Cardiovascular Function Are Major Determinants of Endotoxin-Induced Insulin Resistance. Cardiovasc. Diabetol. 14, 56. doi: 10.1186/s12933-015-0223-2 PMC4484635 25986700

[B31] Hwang I. Park Y. J. Kim Y. Kim Y. N. Ka S. Lee H. Y.. (2015). Alteration of Gut Microbiota by Vancomycin and Bacitracin Improves Insulin Resistance *via* Glucagon-Like Peptide 1 in Diet-Induced Obesity. FASEB J. 29, 2397–2411. doi: 10.1096/fj.14-265983 25713030

[B32] Irimia J. M. Meyer C. M. Segvich D. M. Surendran S. Depaoli-Roach A. A. Morral N.. (2017). Lack of Liver Glycogen Causes Hepatic Insulin Resistance and Steatosis in Mice. J. Biol. Chem. 292, 10455–10464. doi: 10.1074/jbc.M117.786525 28483921 PMC5481557

[B33] Isah M. B. Masola B. (2017). Effect of Oleanolic Acid on Small Intestine Morphology and Enzymes of Glutamine Metabolism in Diabetic Rats. Int. J. Physiol. Pathophysiol. Pharmacol. 9, 128–136. 29209449 PMC5698689

[B34] Jernberg C. Löfmark S. Edlund C. Jansson J. K. (2010). Long-Term Impacts of Antibiotic Exposure on the Human Intestinal Microbiota. Microbiology 156, 3216–3223. doi: 10.1099/mic.0.040618-0 20705661

[B35] Jiang X. Ellabaan M. M. H. Charusanti P. Munck C. Blin K. Tong Y.. (2017). Dissemination of Antibiotic Resistance Genes From Antibiotic Producers to Pathogens. Nat. Commun. 8 (15784). doi: 10.1038/ncomms15784 PMC5467266 28589945

[B36] Kakimoto P. A. Chausse B. Caldeira da Silva C. C. Donato Júnior J. Kowaltowski A. J. (2019). Resilient Hepatic Mitochondrial Function and Lack of iNOS Dependence in Diet-Induced Insulin Resistance. PloS One 14, e0211733. doi: 10.1371/journal.pone.0211733 30716103 PMC6361450

[B37] Kanuri B. N. Kanshana J. S. Rebello S. C. Pathak P. Gupta A. P. Gayen J. R.. (2017). Altered Glucose and Lipid Homeostasis in Liver and Adipose Tissue Pre-Dispose Inducible NOS Knockout Mice to Insulin Resistance. Sci. Rep. 7, 41009. doi: 10.1038/srep41009 28106120 PMC5247703

[B38] Kanuri B. N. Rebello S. C. Pathak P. Agarwal H. Kanshana J. S. Awasthi D.. (2018). Glucose and Lipid Metabolism Alterations in Liver and Adipose Tissue Pre-Dispose P47 ^Phox^ Knockout Mice to Systemic Insulin Resistance. Free Radic. Res. 52, 568–582. doi: 10.1080/10715762.2018.1453136 29544378

[B39] Kim T. Holleman C. L. Ptacek T. Morrow C. D. Habegger K. M. (2017). Duodenal Endoluminal Barrier Sleeve Alters Gut Microbiota of ZDF Rats. Int. J. Obes. 41, 381–389. doi: 10.1038/ijo.2016.224 PMC5340580 27924082

[B40] Kim S. W. Park K. Y. Kim B. Kim E. Hyun C. K. (2013). Lactobacillus Rhamnosus GG Improves Insulin Sensitivity and Reduces Adiposity in High-Fat Diet-Fed Mice Through Enhancement of Adiponectin Production. Biochem. Biophys. Res. Commun. 431, 258–263. doi: 10.1016/j.bbrc.2012.12.121 23313485

[B41] Korpela K. Zijlmans M. A. Kuitunen M. Kukkonen K. Savilahti E. Salonen A.. (2017). Childhood BMI in Relation to Microbiota in Infancy and Lifetime Antibiotic Use. Microbiome 5 (26). doi: 10.1186/S40168-017-0245-Y PMC5335838 28253911

[B42] Kröncke K. D. Fehsel K. Kolb-Bachofen V. (1998). Inducible Nitric Oxide Synthase in Human Diseases. Clin. Exp. Immunol. 113, 147–156. doi: 10.1046/j.1365-2249.1998.00648.x 9717962 PMC1905037

[B43] Kumar A. Kumar Y. Sevak J. K. Kumar S. Kumar N. Gopinath S. D. (2020). Metabolomic Analysis of Primary Human Skeletal Muscle Cells During Myogenic Progression. Sci. Rep. 10, 11824. doi: 10.1038/s41598-020-68796-4 32678274 PMC7366914

[B44] Kuno T. Hirayama-Kurogi M. Ito S. Ohtsuki S. (2018). Reduction in Hepatic Secondary Bile Acids Caused by Short-Term Antibiotic-Induced Dysbiosis Decreases Mouse Serum Glucose and Triglyceride Levels. Sci. Rep. 8. doi: 10.1038/s41598-018-19545-1 PMC5775293 29352187

[B45] Li Z. Agellon L. B. Allen T. M. Umeda M. Jewell L. Mason A.. (2006). The Ratio of Phosphatidylcholine to Phosphatidylethanolamine Influences Membrane Integrity and Steatohepatitis. Cell Metab. 3, 321–331. doi: 10.1016/J.CMET.2006.03.007 16679290

[B46] Liu Y.-X. Cao Q.-M. Ma B.-C. (2019). Pathogens Distribution and Drug Resistance in Patients With Acute Cerebral Infarction Complicated With Diabetes and Nosocomial Pulmonary Infection. BMC Infect. Dis. 19, 1–6. doi: 10.1186/S12879-019-4142-9 31291896 PMC6617900

[B47] Liu L. Zhao J. Zhang R. Wang X. Wang Y. Chen Y.. (2021). Serum Untargeted Metabolomics Delineates the Metabolic Status in Different Subtypes of Non-Alcoholic Fatty Liver Disease. J. Pharm. Biomed. Anal. 200, 114058. doi: 10.1016/J.JPBA.2021.114058 33865049

[B48] Li X. Wang E. Yin B. Fang D. Chen P. Wang G.. (2017b). Effects of Lactobacillus Casei CCFM419 on Insulin Resistance and Gut Microbiota in Type 2 Diabetic Mice. Benef. Microbes 8, 421–432. doi: 10.3920/BM2016.0167 28504567

[B49] Li J. Yang K. Ju T. Ho T. McKay C. A. Gao Y.. (2017a). Early Life Antibiotic Exposure Affects Pancreatic Islet Development and Metabolic Regulation. Sci. Rep. 2017 71 7, 1–12. doi: 10.1038/srep41778 PMC5288777 28150721

[B50] Ma D. W. L. Arendt B. M. Hillyer L. M. Fung S. K. McGilvray I. Guindi M.. (2016). Plasma Phospholipids and Fatty Acid Composition Differ Between Liver Biopsy-Proven Nonalcoholic Fatty Liver Disease and Healthy Subjects. Nutr. Diabetes 6, e220–e220. doi: 10.1038/nutd.2016.27 27428872 PMC4973140

[B51] Matziouridou C. Rocha S. C. Haabeth O. A. Rudi K. Carlsen H. Kielland A. (2017). iNOS- and NOX1-Dependent ROS Production Maintains Bacterial Homeostasis in the Ileum of Mice. Nat. Publ. Gr. 11, 774–784. doi: 10.1038/mi.2017.106 29210363

[B52] Mehta H. H. Xiao J. Ramirez R. Miller B. Kim S. J. Cohen P.. (2019). Metabolomic Profile of Diet-Induced Obesity Mice in Response to Humanin and Small Humanin-Like Peptide 2 Treatment. Metabolomics 15, 88. doi: 10.1007/s11306-019-1549-7 31172328 PMC6554247

[B53] Membrez M. Blancher F. Jaquet M. Bibiloni R. Cani P. D. Burcelin R. G.. (2008). Gut Microbiota Modulation With Norfloxacin and Ampicillin Enhances Glucose Tolerance in Mice. FASEB J. 22, 2416–2426. doi: 10.1096/fj.07-102723 18326786

[B54] Möder M. Kießling A. Löster H. Brüggemann L. (2003). The Pattern of Urinary Acylcarnitines Determined by Electrospray Mass Spectrometry: A New Tool in the Diagnosis of Diabetes Mellitus. Anal. Bioanal. Chem. 375, 200–210. doi: 10.1007/S00216-002-1654-7 12560963

[B55] Nakata S. Tsutsui M. Shimokawa H. Suda O. Morishita T. Shibata K.. (2008). Spontaneous Myocardial Infarction in Mice Lacking All Nitric Oxide Synthase Isoforms. Circulation 117, 2211–2223. doi: 10.1161/CIRCULATIONAHA.107.742692 18413498

[B56] Nobel Y. R. Cox L. M. Kirigin F. F. Bokulich N. A. Yamanishi S. Teitler I.. (2015). Metabolic and Metagenomic Outcomes From Early-Life Pulsed Antibiotic Treatment. Nat. Commun. 6, 7486. doi: 10.1038/ncomms8486 26123276 PMC4491183

[B57] Nowak C. Hetty S. Salihovic S. Castillejo-Lopez C. Ganna A. Cook N. L.. (2018). Glucose Challenge Metabolomics Implicates Medium-Chain Acylcarnitines in Insulin Resistance. Sci. Rep. 2018 81 8, 1–10. doi: 10.1038/s41598-018-26701-0 PMC5989236 29875472

[B58] Nozaki Y. Fujita K. Wada K. Yoneda M. Kessoku T. Shinohara Y.. (2015). Deficiency of iNOS-derived NO Accelerates Lipid Accumulation-Independent Liver Fibrosis in Non-alcoholic Steatohepatitis Mouse Model. BMC Gastroenterology 15, 42. doi: 10.1186/s12876-015-0269-3 PMC4387704 25881230

[B59] Ormazabal V. Nair S. Elfeky O. Aguayo C. Salomon C. Zuñiga F. A. (2018). Association Between Insulin Resistance and the Development of Cardiovascular Disease. Cardiovasc. Diabetol. 17, 122. doi: 10.1186/s12933-018-0762-4 30170598 PMC6119242

[B60] Otsuka M. Kang Y. J. Ren J. Jiang H. Wang Y. Omata M.. (2010). Distinct Effects of P38α Deletion in Myeloid Lineage and Gut Epithelia in Mouse Models of Inflammatory Bowel Disease. Gastroenterology 138, 1255. doi: 10.1053/j.gastro.2010.01.005 20080092 PMC2846963

[B61] Park K.-Y. Kim B. Hyun C.-K. (2015). Lactobacillus Rhamnosus GG Improves Glucose Tolerance Through Alleviating ER Stress and Suppressing Macrophage Activation in Db/Db Mice. J. Clin. Biochem. Nutr. 56, 240–246. doi: 10.3164/jcbn.14-116 26060355 PMC4454087

[B62] Pathak P. Kanshana J. S. Kanuri B. Rebello S. C. Aggarwal H. Jagavelu K.. (2019). Vasoreactivity of Isolated Aortic Rings From Dyslipidemic and Insulin Resistant Inducible Nitric Oxide Synthase Knockout Mice. Eur. J. Pharmacol. 855, 90–97. doi: 10.1016/j.ejphar.2019.05.005 31063772

[B63] Perreault M. Marette A. (2001). Targeted Disruption of Inducible Nitric Oxide Synthase Protects Against Obesity-Linked Insulin Resistance in Muscle. Nat. Med. 7, 1138–1143. doi: 10.1038/nm1001-1138 11590438

[B64] Pushpanathan P. Srikanth P. Seshadri K. G. Selvarajan S. Pitani R. S. Kumar T. D.. (2016). Gut Microbiota in Type 2 Diabetes Individuals and Correlation With Monocyte Chemoattractant Protein1 and Interferon Gamma From Patients Attending a Tertiary Care Centre in Chennai, India. Indian J. Endocrinol. Metab. 20, 523. doi: 10.4103/2230-8210.183474 27366720 PMC4911843

[B65] Radilla-Vázquez R. B. Parra-Rojas I. Martínez-Hernández N. E. Márquez-Sandoval Y. F. Illades-Aguiar B. Castro-Alarcón N. (2016). Gut Microbiota and Metabolic Endotoxemia in Young Obese Mexican Subjects. Obes. Facts 9, 1. doi: 10.1159/000442479 PMC5644836 26745497

[B66] Ravussin Y. Koren O. Spor A. Leduc C. Gutman R. Stombaugh J.. (2012). Responses of Gut Microbiota to Diet Composition and Weight Loss in Lean and Obese Mice. Obesity 20, 738–747. doi: 10.1038/oby.2011.111 21593810 PMC3871199

[B67] Rawat V. Singhai M. Kumar A. Jha P. K. Goyal R. (2012). Bacteriological and Resistance Profile in Isolates From Diabetic Patients. N. Am. J. Med. Sci. 4, 563. doi: 10.4103/1947-2714.103315 23181227 PMC3503374

[B68] Ray P. Pandey U. Aich P. (2021). Comparative Analysis of Beneficial Effects of Vancomycin Treatment on Th1- and Th2-Biased Mice and the Role of Gut Microbiota. J. Appl. Microbiol 130, 1337–1356. doi: 10.1111/jam.14853 32955795

[B69] Roager H. M. Licht T. R. (2018). Microbial Tryptophan Catabolites in Health and Disease. Nat. Commun. 9, 3294. doi: 10.1038/s41467-018-05470-4 30120222 PMC6098093

[B70] Rodrigues R. R. Greer R. L. Dong X. DSouza K. N. Gurung M. Wu J. Y.. (2017). Antibiotic-Induced Alterations in Gut Microbiota Are Associated With Changes in Glucose Metabolism in Healthy Mice. Front. Microbiol. 8, 2306. doi: 10.3389/fmicb.2017.02306 29213261 PMC5702803

[B71] Scheiman J. Luber J. M. Chavkin T. A. MacDonald T. Tung A. Pham L. D.. (2019). Meta-Omics Analysis of Elite Athletes Identifies a Performance-Enhancing Microbe That Functions *via* Lactate Metabolism. Nat. Med. 25, 1104–1109. doi: 10.1038/s41591-019-0485-4 31235964 PMC7368972

[B72] Shapiro H. Suez J. Elinav E. (2017). Personalized Microbiome-Based Approaches to Metabolic Syndrome Management and Prevention. J. Diabetes 9, 226–236. doi: 10.1111/1753-0407.12501 27787945

[B73] Stecher B. Denzler R. Maier L. Bernet F. Sanders M. J. Pickard D. J.. (2012). Gut Inflammation can Boost Horizontal Gene Transfer Between Pathogenic and Commensal Enterobacteriaceae. Proc. Natl. Acad. Sci. U. S. A. 109, 1269–1274. doi: 10.1073/pnas.1113246109 22232693 PMC3268327

[B74] Sullivan Å. (2001). Effect of Antimicrobial Agents on the Ecological Balance of Human Microflora. Lancet Infect. Dis. 1, 101–114. doi: 10.1016/S1473-3099(01)00066-4 11871461

[B75] Tandon D. Haque M. M. Saravanan R. Shaikh S. Sriram P. Dubey A. K.. (2018). A Snapshot of Gut Microbiota of an Adult Urban Population From Western Region of India. PloS One 13, e0195643. doi: 10.1371/journal.pone.0195643 29624599 PMC5889170

[B76] Thevaranjan N. Puchta A. Schulz C. Naidoo A. Szamosi J. C. Verschoor C. P.. (2017). Age-Associated Microbial Dysbiosis Promotes Intestinal Permeability, Systemic Inflammation, and Macrophage Dysfunction. Cell Host Microbe 21, 455–466.e4. doi: 10.1016/j.chom.2017.03.002 28407483 PMC5392495

[B77] Tulstrup M. V.-L. Christensen E. G. Carvalho V. Linninge C. Ahrné S. Højberg O.. (2015). Antibiotic Treatment Affects Intestinal Permeability and Gut Microbial Composition in Wistar Rats Dependent on Antibiotic Class. PloS One 10, e0144854. doi: 10.1371/journal.pone.0144854 26691591 PMC4686753

[B78] Turnbaugh P. J. Hamady M. Yatsunenko T. Cantarel B. L. Duncan A. Ley R. E.. (2009). A Core Gut Microbiome in Obese and Lean Twins. Nature 457, 480–484. doi: 10.1038/nature07540 19043404 PMC2677729

[B79] Urasaki Y. Pizzorno G. Le T. T. (2016). Chronic Uridine Administration Induces Fatty Liver and Pre-Diabetic Conditions in Mice. PloS One 11. doi: 10.1371/journal.pone.0146994 PMC4720477 26789264

[B80] Wang T. J. Ngo D. Psychogios N. Dejam A. Larson M. G. Vasan R. S.. (2013). 2-Aminoadipic Acid Is a Biomarker for Diabetes Risk. J. Clin. Invest 123, 4309–4317. doi: 10.1172/JCI64801 24091325 PMC3784523

[B81] Wilkins A. T. Reimer R. A. (2021). Obesity, Early Life Gut Microbiota, and Antibiotics. Microorganisms 9, 1–20. doi: 10.3390/MICROORGANISMS9020413 PMC7922584 33671180

[B82] Wong J. M. Billiar T. R. (1995). Regulation and Function of Inducible Nitric Oxide Synthase During Sepsis and Acute Inflammation. Adv. Pharmacol. 34, 155–170. doi: 10.1016/S1054-3589(08)61084-4 8562431

[B83] Yokoyama H. Emoto M. Fujiwara S. Motoyama K. Morioka T. Komatsu M.. (2003). Quantitative Insulin Sensitivity Check Index and the Reciprocal Index of Homeostasis Model Assessment in Normal Range Weight and Moderately Obese Type 2 Diabetic Patients. Diabetes Care 26, 2426–2432. doi: 10.2337/diacare.26.8.2426 12882874

[B84] Zarrinpar A. Chaix A. Xu Z. Z. Chang M. W. Marotz C. A. Saghatelian A.. (2018). Antibiotic-Induced Microbiome Depletion Alters Metabolic Homeostasis by Affecting Gut Signaling and Colonic Metabolism. Nat. Commun. 9, 2872. doi: 10.1038/s41467-018-05336-9 30030441 PMC6054678

[B85] Zhang L. (2015). A Systematic Review of Metabolite Profiling in Diabetic Nephropathy. J. Endocrinol. Diabetes 2, 01–11. doi: 10.15226/2374-6890/2/3/00127

[B86] Zhao Q. Zhang A. Zong W. An N. Zhang H. Luan Y.. (2017). Exploring Potential Biomarkers and Determining the Metabolic Mechanism of Type 2 Diabetes Mellitus Using Liquid Chromatography Coupled to High-Resolution Mass Spectrometry. RSC Adv. 7, 44186–44198. doi: 10.1039/c7ra05722a

[B87] Zhou W. Xu H. Zhan L. Lu X. Zhang L. (2019). Dynamic Development of Fecal Microbiome During the Progression of Diabetes Mellitus in Zucker Diabetic Fatty Rats. Front. Microbiol. 10, 232. doi: 10.3389/fmicb.2019.00232 30837966 PMC6382700

