# Supplementary material for: Vancomycin-Induced Modulation of Gram-Positive Gut Bacteria and Metabolites Remediates Insulin Resistance in iNOS Knockout Mice
Source: Front Cell Infect Microbiol. 2022 Jan 19;11:795333. doi: 10.3389/fcimb.2021.795333 (PMC8807491; doi:10.3389/fcimb.2021.795333)
Supplement: Supplementary file 2 [file DataSheet_2.docx]

Supplementary Material

# Supplementary Figures and Tables

## Supplementary Figures

**Supplementary Figure 1.** **Fig. S1. related to figure 1. Systemic glucose, insulin and lipid homeostasis in iNOS^-/-^ mice.** Systemic IR analysis in WT and iNOS^-/-^ mice. (A) Intraperitoneal glucose tolerance test (GTT), (B) Area under the curve (AUC) calculated from IPGTT data, (C) Fasting blood glucose levels, (D) Fasting serum insulin levels, (E) Index of insulin resistance, HOMA-IR, (F) Intraperitoneal insulin tolerance test (ITT), (G) AUC calculated from ITT, (H) Index of insulin sensitivity, QUCIKI, (I) Serum total cholesterol (TC), (J) Serum triglycerides (TG), (K) Intraperitoneal pyruvate tolerance test (PTT), (L) AUC calculated from PTT, (M) Serum low density lipoprotein (LDL), (N) Serum non-esterified free fatty acids (NEFA) and (O) Serum high density lipoprotein (HDL). Data are represented as mean ± SEM (n≥6). *p<0.05, **p<0.01, ***p<0.001, ****p<0.0001 vs WT.

**Supplementary Figure 2. Fig. S2. related to figure 2 and 3.**

**Vancomycin-induced gut microbiota modulation and associated serum metabolites in WT mice.** Serum metabolomic analysis and gut microbiota analysis in untreated and vancomycin treated WT mice. (A) PCA score plot of metabolomic analysis in untreated and vancomycin treated WT mice in ESI (+) mode. Heat map of differential metabolites found by metabolomics analysis in WT with and without vancomycin treatment related to (B) Vitamins and hormones metabolism, (C) Nucleic acids metabolism, (D) Miscellaneous/ microbiota derived metabolites, (E) PCA plot of gut microbiota analysis in untreated and vancomycin treated WT mice based on Bray-Curtis distance. Heat map of differential metabolites found by metabolomics analysis in WT with and without vancomycin treatment related to (F) Carbohydrate metabolism and (G) Amino acids metabolism. (H) Differentially abundant microbiota at the phylum, family and genus level. (I) Heat map of differential metabolites found by metabolomics analysis in WT with and without vancomycin treatment related to lipid metabolism. Data are represented as mean ± SEM (n≥6). *p<0.05, **p<0.01, ***p<0.001, ****p<0.0001 vs WT.

**Supplementary Figure 3. Fig. S3. related to figure 2.**

**Antibiotic cocktail-induced modulation of gut microbiota rescue iNOS^-/-^ mice from systemic IR and dyslipidemia.** Gut microbiota analysis in untreated and Abx treated iNOS^-/-^ mice. α-diversity indices in stool samples (A) Observed, (B) Shannon, (C) Simpson and (D) Chao1 index. (E) PCA plot based on Bray-Curtis distance. (F) Differentially abundant microbiota at the phylum, family and genus level. Systemic IR analysis in Abx treated and untreated WT and iNOS^-/-^ mice. (G) Intraperitoneal glucose tolerance test (GTT), (H) Area under the curve (AUC) calculated from IPGTT data, (I) Intraperitoneal insulin tolerance test (ITT), (J) AUC calculated from ITT (K) Intraperitoneal pyruvate tolerance test (PTT), (L) AUC calculated from PTT, (M) Fasting blood glucose levels and (N) Fasting serum insulin levels. Indices of insulin sensitivity (O) HOMA-IR and (P) QUCIKI. Serum lipids (Q) Total cholesterol (TC), (R) Triglycerides (TG), (S) Low density lipoprotein (LDL) and (T) Non-esterified free fatty acids (NEFA). Data are represented as mean ± SEM (n≥6). *p<0.05, **p<0.01, ***p<0.001, ****p<0.0001 between indicated groups. ^#^p<0.05, ^##^p<0.01 and ^####^p<0.0001 vs iNOS^-/-^.

**Supplementary Figure 4. Fig. S4. related to figure 3.**

**Antibiotic cocktail-induced alterations in serum metabolites in iNOS^-/-^ mice.** Serum metabolomic analysis in in untreated and Abx treated iNOS^-/-^ mice in ESI (+) mode. (A) PCA score plot and (B) Volcano plot of differential metabolites between iNOS^-/-^ mice with or without Abx treatment. Red in volcano plot indicates significantly up-regulated metabolites, green indicates the down-regulated metabolites, and grey shows no significant difference. Heat map of differential metabolites found by metabolomics analysis in iNOS^-/-^ mice with and without Abx treatment related to (C) Miscellaneous/ microbiota derived metabolites, (D) Carbohydrate metabolism, (E) Amino acid metabolism, (F) Vitamins, hormones and bile acids metabolism, (G) Lipid metabolism and (H) Nucleic acids metabolism. Data are represented as mean ± SEM (n≥6). *p<0.05, **p<0.01, ***p<0.001, ****p<0.0001 vs iNOS^-/-^.

**upplementary Figure 5. Fig. S5. related to figure 2 and 3.**

**Antibiotic cocktail-induced gut microbiota modulation and associated serum metabolites in WT mice.** Serum metabolomic analysis and gut microbiota analysis in untreated and Abx treated WT mice. (A) PCA score plot of metabolomic analysis in untreated and Abx treated WT mice in ESI (+) mode. Heat map of differential metabolites found by metabolomics analysis in WT with and without Abx treatment related to (B) Vitamins, hormones and bile acids metabolism, (C) Carbohydrate metabolism, (D) Nucleic acids metabolism, (E) Miscellaneous/ microbiota derived metabolites. (F) PCA plot of gut microbiota analysis in untreated and Abx treated WT mice based on Bray-Curtis distance. (G) Heat map of differential metabolites found by metabolomics analysis in WT with and without Abx treatment related to amino acids metabolism. (H) Differentially abundant microbiota at the phylum, family and genus level. (I) Heat map of differential metabolites found by metabolomics analysis in WT with and without Abx treatment related to lipid metabolism. Data are represented as mean ± SEM (n≥6). *p<0.05, **p<0.01, ***p<0.001, ****p<0.0001 vs WT.

**Supplementary Figure 6. Fig. S6. related to figure 2.**

**Effect of antibiotics on gross parameters in insulin resistant iNOS^-/-^ mice.** (A) Body weight curve from the initiation (0 week) to study termination (4 weeks), (B) Area under the curve (AUC) calculated from the gradual change in the body weight, (C) Whole body fat mass and lean mass (%), (D) Weekly food consumption from study initiation (0 week) to study termination (4 weeks) and (E) Average food consumption per day during the study period, (F) Body mass index (BMI), (G) Relative tissue weights and (H) Relative intestine length. Data are represented as mean ± SEM (n≥6). *p<0.05, **p<0.01, ***p<0.001 and ****p<0.0001 between indicated groups and *p<0.05, **p<0.01 vs WT in the body weight curve.

**Supplementary Figure 7. Fig. S7. related to figure 4.**

**Improvement in the disrupted lipid and glucose homeostasis in liver in iNOS^-/-^ mice following treatment with antibiotic cocktail.** Hepatic lipid levels in WT and iNOS^-/-^ mice with and without Abx treatment (A) TC, (B) TG and (C) FFA. (D) Hepatic glycogen levels with or without insulin stimulation in WT and iNOS^-/-^ mice with and without Abx treatment. Hepatic mRNA expression analysis of genes involved in (E) Gluconeogenesis, (F) Lipid synthesis, (G) Lipid oxidation, (H) Lipid uptake and (I) Lipid efflux. Data are represented as mean ± SEM (n≥6). *p<0.05, **p<0.01, ***p<0.001, ****p<0.0001 between indicated groups.

**Supplementary Figure 8. Fig. S8. related to figure 5.**

**Improvement in the disrupted lipid and glucose homeostasis in adipose tissue and intestine in iNOS^-/-^ mice following treatment with antibiotic cocktail.** Adipose tissue mRNA expression analysis of genes involved in (A) Gluconeogenesis, (B) Glucose homeostasis, (C) Lipid synthesis, (D) Lipid oxidation and (E) Lipid uptake. Intestinal tissue mRNA expression analysis of genes involved in (F) Lipid synthesis, (G) Lipid uptake and (H) Lipid efflux. Data are represented as mean ± SEM (n≥6). *p<0.05, **p<0.01, ***p<0.001, ****p<0.0001 between indicated groups.

**Supplementary Figure 9. Fig. S9. related to figure 5.**

**Gut barrier functionality and NO homeostasis in the insulin resistant iNOS^-/-^ mice upon antibiotics treatment.** Histological analysis (A) Small intestinal tissue sections stained with Alcian blue-nuclear fast red for detection of goblet cells and morphology, (B) Percent Alcian blue stained area in small intestinal tissue and (C) Intestinal villi length. Histological analysis (D) Colonic tissue sections stained with Alcian blue-nuclear fast red for detection of goblet cells and morphology, (E) % Alcian blue stained area in colon tissue and (F) Colonic crypt length. (G) Intestinal permeability as measured via FITC-dextran oral gavage assay showing FITC-dextran concentration in serum. (H) mRNA expression analysis of tight junctions, mucins and antimicrobial peptide in small intestine. Total nitrite levels in (I) Serum, (J) Liver, (K) Adipose tissue and (L) Intestine. NOS isoforms mRNA expression analysis in (M) Liver, (N) White adipose tissue and (O) Small intestine. Scale bars, 100 µm. Data are represented as mean ± SEM (n≥6). *p<0.05, **p<0.01, ***p<0.001, ****p<0.0001 between indicated groups.

**Supplementary Figure 10. Fig. S10.**

**Association of serum metabolites with metabolic profile of iNOS^-/-^ mice upon treatment with antibiotics.** Heat map of correlation analysis between metabolic biomarkers and serum metabolites in WT and iNOS^-/-^ mice treated with antibiotics- vancomycin or Abx along with untreated controls based on Pearson’s correlation coefficients related to (A) lipid metabolism, (B) amino acid metabolism, (C) Nucleic acids and carbohydrate metabolism, vitamins, cofactors, hormones and bile acids, (D) Miscellaneous/ microbiota derived metabolites. *p<0.05 represent significant correlations between metabolic biomarker and serum metabolite. Green color represents negative and red positive correlations.

**Supplementary Figure 11. Fig. S11. related to figure 6.**

**Association of altered gut microbiome by antibiotics with metabolic parameters in iNOS^-/-^ mice.** Heat map of Pearson’s correlation coefficients between changes in different metabolic parameters and taxa at family level caused by gut microbiota modulation by vancomycin and Abx in WT and iNOS^-/-^ mice. *p<0.05, **p<0.01, represent significant correlations between metabolic biomarker and bacterial taxa. Blue color represents negative and red positive correlations.

**Supplementary Figure 12. Fig. S12.**

**Gut microbiota association with serum metabolites in iNOS^-/-^ mice upon antibiotics treatment.** Heat map of correlation analysis between gut microbiota and serum metabolites related to (A) Lipid metabolism, (B) Amino acids metabolism, (C) Miscellaneous/ microbiota derived metabolites and (D) Cofactors, hormones, bile acids, carbohydrate and nucleic acid metabolites in WT and iNOS^-/-^ mice treated with antibiotics- vancomycin or Abx along with untreated controls based on Pearson’s correlation coefficients. *p<0.0 represent significant correlations between bacterial taxa and serum metabolite. Blue color represents negative and orange positive correlations.

## Supplementary Tables

**Table S1: Primer sequences used for gene expression analysis using qPCR.**

| **Gene** | **Forward primer sequence (5'-3')** | **Reverse primer sequence (5'-3')** |
| --- | --- | --- |
| **NOS isoforms** | | |
| **eNOS** | CAACGCTACCACGAGGACATT | CTCCTGCAAAGAAAAGCTCTGG |
| **nNOS** | CCAACCCAACGTCATTTCTG | CATAGCTGAGGTCTACCAGG |
| **Gluconeogenesis** | | |
| **PEPCK** | TCTCTGATCCAGACCTTCCAA | GAAGTCCAGACCGTTATGCAG |
| **G6PC** | AAGCCAACGTATGGATTCCG | ACAGCAATGCCTGACAAGACT |
| **PC** | GGGATGCCCACCAGTCACT | CATAGGGCGCAATCTTTTTGA |
| **FOXO1** | AAGAGCGT GCCCTACTTCAA | TGCT GT GAAGGGACAGATTG |
| **Glucose homeostasis** | | |
| **Glut 2** | CCCTGGGTACTCTTCACCAA | GCCAAGTAGGATGTGCCAAT |
| **Glut 4** | AAAAGTGCCTGAAACCAGAG | TCACCTCCTGCTCTAAAAGG |
| **Akt2** | AAAAAGTGGCTCTGGTGTGTG | GGCATTCTGCTACAGAGAAATTG |
| **Lipid homeostasis** | | |
| **SREBP-1c** | GGAGCCATGGATTGCACATT | CCTGTCTCACCCCCAGCATA |
| **SREBP-2** | GCGTTCTGGAGACCATGGA | ACAAAGTTGCTCTGAAAACAAATCA |
| **FAS** | GGCATCATTGGGCACTCCTT | GCTGCAAGCACAGCCTCTCT |
| **ACC1** | GGACAGACTGATCGCAGAGAAAG | TGGAGAGCCCCACACACA |
| **HMGCR** | CTTGTGGAATGCCTTGTGATTG | AGCCGAAGCAGCACATGAT |
| **PPARγ** | AGTGGAGACCGCCCAGG | GCAGCAGGTTGTCTTGGATGT |
| **LXRα** | GCTCTGCTCATTGCCATCAG | TGTTGCAGCCTCTCTACTTGGA |
| **LXRβ** | AAGCAGGTGCCAGGGTTCT | TGCATTCTGTCTCGTGGTTGT |
| **ACC2** | GGGCTCCCTGGATGACAAC | GCTCTTCCGGGAGGAGTTCT |
| **PPARα** | GTCCTCAGTGCTTCCAGAGG | GGTCACCTACGAGTGGCATT |
| **PGC-1α** | AACCACACCCACAGGATCAGA | TCTTCGCTTTATTGCTCCATGA |
| **PGC-1β** | CGCTCCAGGAGACTGAATCCAG | CTTGACTACTGTCTGTGAGGC |
| **UCP2** | CAGCCAGCGCCCAGTACC | CAATGCGGACGGAGGCAAAGC |
| **CYP7A1** | GCTAAGACGCACCTCGTGAT | AGGGCTCCTGATCATTTGAA |
| **Lipid uptake and lipolysis** | | |
| **CD36/FAT** | GCAAAACGACTGCAGGTCAAC | TGGTCCCAGTCTCATTTAGCCA |
| **FABP1** | TGCACCACCAACTGCTTAGC | AGCTCAGGGATGA CCTTGCC |
| **SR-1B** | GGCTGCTGTTTGCTGCG | GCTGCTTGATGAGGGAGGG |
| **ApoE** | AACCGCTTCTGGGATTACCT | CAGTGCCGTCAGTTCTTGTG |
| **LPL** | AAGGTCAGAGCCAAGAGAAGCA | CCAGAAAAGTGAATCTTGACTTGGT |
| **LDLR** | GCATCAGCTTGGACAAGGTGT | GGGAACAGCCACCATTGTTG |
| **NPC1L1** | TTGCCTTGACCTCTGGCTTAG | AGGGCGGATGAATCTGTGC |
| **FFAR1** | TGGCTAGTTTCATAAACCCGG | TCCCAAGTAGCCATGGACCAGT |
| **FFAR2** | TGTTCAGTTCCCTCAATGCCA | CAGGATTGCGGATCAGTAGCA |
| **Lipid efflux** | | |
| **ABCG5** | TGGATCCAACACCTCTATGCTAAA | GGCAGGTTTTCTCGATGAACTG |
| **ABCG8** | TGCCCACCTTCCACATGTC | ATGAAGCCGGCAGTAAGGTAGA |
| **ABCA1** | CGTTTCCGGGAAGTGTCCTA | GCTAGAGATGACAAGGAGGATGGA |
| **Intestinal homeostasis** | | |
| **Reg3γ** | TTCCTGTCCTCCATGATCAAA | CATCCACCTCTGTTGGGTTC |
| **Occludin** | ATGTCCGGCCGATGCTCTC | TTTGGCTGCTCTTGGGTCTGTAT |
| **Claudin-2** | GTCATCGCCCATCAGAAGAT | ACTGTTGGACAGGGAACCAG |
| **ZO-1** | ACCCGAAACTGATGCTGTGGATAG | AAATGGCCGGGCAGAACTTGTGTA |
| **Mucin-2** | GGGAGGGTGGAAGTGGCATTGT | TGCTGGGGTTTTTGTGAATCTC |
| **Mucin-5AC** | CACCATCTCTACAACCCAAACT | TGAGGTCCAGGTCTTTGTGTCT |
| **Housekeeping genes** | | |
| **18S rRNA** | GCAATTATTCCCCATGAACG | GGCCTCACTAAACCATCCAA |
| **RPLP0** | ACCTCCTTCTTCCAGGCTTT | CCCACCTTGTCTCCAGTCTTT |

**Table S2:** Sequencing stats

| **Sample Name** | **Total reads** | **Processed reads** | **Total Identified rRNA sequences** | **Total OTUs Picked** |
| --- | --- | --- | --- | --- |
| 1. WT | 183209 | 179415 | 108806 | 842 |
| 2. WT | 128148 | 125655 | 76787 | 729 |
| 3. WT | 206183 | 201920 | 106451 | 931 |
| 4. WT | 137362 | 135001 | 71240 | 893 |
| 5. WT | 168549 | 164574 | 80460 | 947 |
| 6. WT | 115264 | 113046 | 67265 | 842 |
| 7. iNOS^-/-^ | 114718 | 112334 | 68925 | 690 |
| 8. iNOS^-/-^ | 173539 | 169936 | 104054 | 743 |
| 9. iNOS^-/-^ | 164919 | 161525 | 86904 | 741 |
| 10. iNOS^-/-^ | 114677 | 112281 | 63174 | 669 |
| 11. iNOS^-/-^ | 147363 | 144329 | 81940 | 827 |
| 12. iNOS^-/-^ | 178833 | 174822 | 97976 | 753 |
| 13. WT+VANCO | 110142 | 108088 | 75980 | 228 |
| 14. WT+VANCO | 195475 | 192061 | 131604 | 331 |
| 15. WT+VANCO | 249801 | 244649 | 139247 | 340 |
| 16. WT+VANCO | 140967 | 138144 | 90258 | 388 |
| 17. WT+VANCO | 248921 | 244438 | 141474 | 367 |
| 18. WT+VANCO | 123200 | 121505 | 83789 | 390 |
| 19. iNOS^-/-^+VANCO | 165779 | 162597 | 116176 | 384 |
| 20. iNOS^-/-^+VANCO | 149928 | 147018 | 106807 | 211 |
| 21. iNOS^-/-^+VANCO | 105304 | 103130 | 73856 | 238 |
| 22. iNOS^-/-^+VANCO | 171297 | 168081 | 114233 | 421 |
| 23. iNOS^-/-^+VANCO | 117734 | 115572 | 81010 | 391 |
| 24. iNOS^-/-^+VANCO | 140438 | 137929 | 99579 | 194 |
| 25. WT+ABX | 78137 | 76900 | 38725 | 668 |
| 26. WT+ABX | 148377 | 145930 | 100182 | 702 |
| 27. WT+ABX | 179441 | 176414 | 117977 | 353 |
| 28. WT+ABX | 121543 | 119378 | 60330 | 569 |
| 29. WT+ABX | 90882 | 89617 | 49942 | 455 |
| 30. WT+ABX | 95316 | 93903 | 48703 | 462 |
| 31. iNOS^-/-^+ABX | 134522 | 132311 | 100375 | 336 |
| 32. iNOS^-/-^+ABX | 123028 | 121523 | 96390 | 280 |
| 33. iNOS^-/-^+ABX | 107353 | 104816 | 73534 | 302 |
| 34. iNOS^-/-^+ABX | 119397 | 117939 | 87394 | 614 |
| 35. iNOS^-/-^+ABX | 89273 | 88012 | 69079 | 172 |
| 36. iNOS^-/-^+ABX | 108082 | 106288 | 77976 | 395 |

**Excel Table S3:** Absolute reads count supporting the particular bacterial species/genus/family/phylum
